# Liposome encapsulated surfactant abetted copper nanoparticles alleviates biofilm mediated virulence in pathogenic *Pseudomonas aeruginosa* and MRSA

**DOI:** 10.1038/s41598-020-79976-7

**Published:** 2021-01-13

**Authors:** Suganya Kannan, Anitta Solomon, Govindan Krishnamoorthy, Murugan Marudhamuthu

**Affiliations:** Department of Microbial Technology, School of Biological Sciences, Madurai Kamaraj University, Tamil Nadu, Madurai, 625021 India

**Keywords:** Antimicrobials, Bacteria, Clinical microbiology

## Abstract

In the present study lipopeptide biosurfactant with high emulsification capacity produced by human skin bacterium *Paenibacillus thiaminolyticus* was purified and subjected to FTIR and NMR spectral analysis which gave evidence of the active characteristics of the surfactant. To augment the antivirulent potential further, the mixer of copper and copper oxide nanoparticles (CuNPs) was synthesized, and characterized by UV–Visible spectroscopy, SEM-EDAX, TEM, and Zeta analysis. Here, we attempted to enhance the antimicrobial and antibiofilm activity with the assistance of encapsulated preparation of lipopeptide and CuNPs in multilamellar liposomes. The proposed mechanism of action of lipopeptide and CuNPs liposomal preparation negatively influences the cell metabolism, secreted virulence such as staphyloxanthin, pyocyanin, and extracellular polysaccharides. The significant decline in the growth of MRSA and *P. aeruginosa* in both planktonic form and biofilm by lipopeptide and CuNPs treatment were visualized using scanning electron microscopy and High content screening imaging system. In vivo studies revealed that treatment with lipopeptide and CuNPs in multilamellar liposomes extended the lifespan of infected *Caenorhabditis elegans* by about 75%. Therefore, this study typifies lipopeptide and CuNPs could credibly be a substantial substitute over conventional antibiotics in averting the biofilm associated pathogenesis of MRSA and *P. aeruginosa*.

## Introduction

Finding of non-antibiotic substitutions to combat severe infections instigated by multidrug-resistant (MDR) bacterial communities represents the top priorities in healthcare settings^1^. The ESKAPE pathogens (*Enterococcus faecium, Staphylococcus aureus, Klebsiella pneumoniae, Acinetobacter baumannii, Pseudomonas aeruginosa, and Enterobacter species*) categorized under a significant group of universally infecting Multi-Drug Resistant pathogens at the heart of the antibiotic resistance calamity^2^. The most prominent virulent members in the group of ESKAPE pathogens are *Pseudomonas aeruginosa* and methicillin-resistant *S. aureus* (MRSA)^3^. These pathogens endorse biofilm facilitated infections which cause augmented resistance to antimicrobial drugs, frequently necessitating therapeutic doses several orders of extent greater than those required to eliminate planktonic cells^4^. These pathogens develop detrimental biofilms on expedients, urinary, endotracheal, intravenous, and other forms of catheters and implants introduced into more than 25% of patients in the course of hospitalization^5^.

*P. aeruginosa* causes severe foodborne and nosocomial infections including pneumonia, terminal burn infections, urinary tract infection, and sepsis. The infections by *P. aeruginosa* are tough to exterminate due to its possible virulence factors such as pyocyanin, exopolysaccharides, secretary proteases, and hemolysin. Methicillin-resistant *Staphylococcus aureus* (MRSA) causes infections such as catheter-related bloodstream infections (CRBSI), wound infections, healthcare, and community-associated infections^6^. MRSA limiting the therapeutic options by the expression of virulence genes, cell to cell signaling mechanism, inactivation of antibiotics, and alteration in target sites, efflux pumps, and biofilm formation. A consequence of critical diseases is the augmented number of chronic *P. aeruginosa* and MRSA infections due to the dominance of biofilm colonization^7^. These biofilm embedded bacterial cells can be 1,000 times more resistant to antibiotics.

Biosurfactants especially lipopeptide classes are amphipathic natural surfactants synthesized by a variety of bacteria. Recently, there has been more attention paid to the biosurfactants from human microbial flora is often documented as a defense weapon against infectious bacteria colonizing in the skin, gastrointestinal, oral and urogenital tract^8^. The biosurfactants produced by *Lactobacillus acidophilus* inhibit the adherence of *Listeria monocytogenes* to stainless steel surfaces, and biosurfactant from *Lactobacillus fermentum* inhibits the adherence of *Enterococcus faecalis*^9^. However, combinatorial therapy with biosurfactant will be useful in refining antimicrobial efficiency, providing broad-spectrum exposure, and inhibiting the emergence of resistance^10^.

One promising approach in the ground of antibacterial treatment is the application of nanotechnology-assisted components for averting and treating infections caused by resistant bacteria. Nanoparticles exhibit greater antimicrobial potential through the striking mechanisms such as (1) attachment towards bacterial superficial area, (2) deterioration of bacterial cell wall and outer membrane with alteration in its penetrability, (3) initiation of toxicity and oxidative stress by ROS release, and (4) intonation of signal transduction pathways^11^. Nanoparticles sustained on numerous suitable materials, such as carbon, polyurethane foam, polymers, and sepiolite have also been efficiently applied for bactericidal applications.

Based on previous studies, compared with other metal nanoparticles copper releasing Cu^2+^ ions, which eventually leads to the penetration and disruption of the cell membrane and biochemical pathway by chelating cellular enzymes and DNA damage. Currently, copper has been itemized as the chief and only metal with antimicrobial properties by the American Environmental Protection Agency (EPA). Also, in some cases, copper based nanomaterials owns properties superior comparative to the other expensive metals with antimicrobial activity, such as, silver and gold. Despite their ample applicability antimicrobial activity of antibiotics combinations, mainly more than one agent combined, is rarely evaluated in in vitro assays.

To add rigorous information to this field, the current study has intended to formulate multilamellar liposomes encapsulating both biosurfactant and copper nanoparticle in lipid multilayer. To our knowledge, very little information is currently available about how biosurfactants and nanoparticles abetted liposomes exploits the pathogenic microbial biofilms. Therefore, this study aimed to monitor the effect of biosurfactant and CuNPs encapsulated liposomal preparation on MRSA, *P. aeruginosa* growth, and virulence profile.

## Results

**Figure 1:**
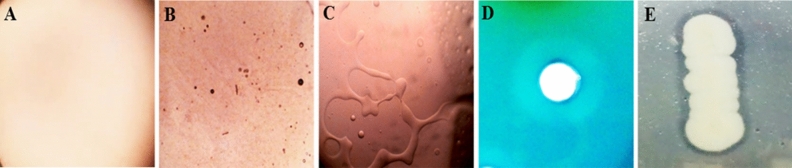
Biosurfactant screening assays (**A**) Oil collapsation test—Control oil droplets without biosurfactant treatment, (**B**) droplet collapsation of oil after the addition of biosurfactant at 20 s, (**C**) at 40 s (10X magnification), (**D**) CTAB- methylene blue zone of biosurfactant production, (**E**) Hydrolysis of tributyrin.

A total of 68 morphologically divergent bacterial isolates were obtained from the skin swabs of 20 healthy individuals. Based on the survival and growth in the media with acid (pH 4.5) and human cathelicidin LL-37, 27 isolates were chosen and screened for biosurfactant production. The isolate SK10 was selected based on the higher emulsification index (81%) showed progressive results in screening assays such as, drop collapse test, CTAB assay, and lipase production (Fig. 1). In the drop collapsing test, a flat drop was spotted within 1 min of the incubation. The distinguished zone was observed from the CTAB assay and the lipase test indicated the biosurfactant production. Isolate SK10 achieved the highest overall surface tension reducing property of 21.3 mN/m from 45 mN/m with the critical micelle concentration of 0.6 mg/ml. Taxonomic association based on the 16S rRNA sequencing data of the isolate SK10 was examined by a mega BLAST tool of GenBank. Based on the closet matches with the strain SK10 was taxonomically identified as *Paenibacillus thiaminolyticus* SK10 . The phylogenetic affiliation was performed by MEGA 6.0 and the sequence obtained was deposited in Genbank with accession number MN549337.1.

### Production, purification, and characterisation of biosurfactant

**Figure 2:**
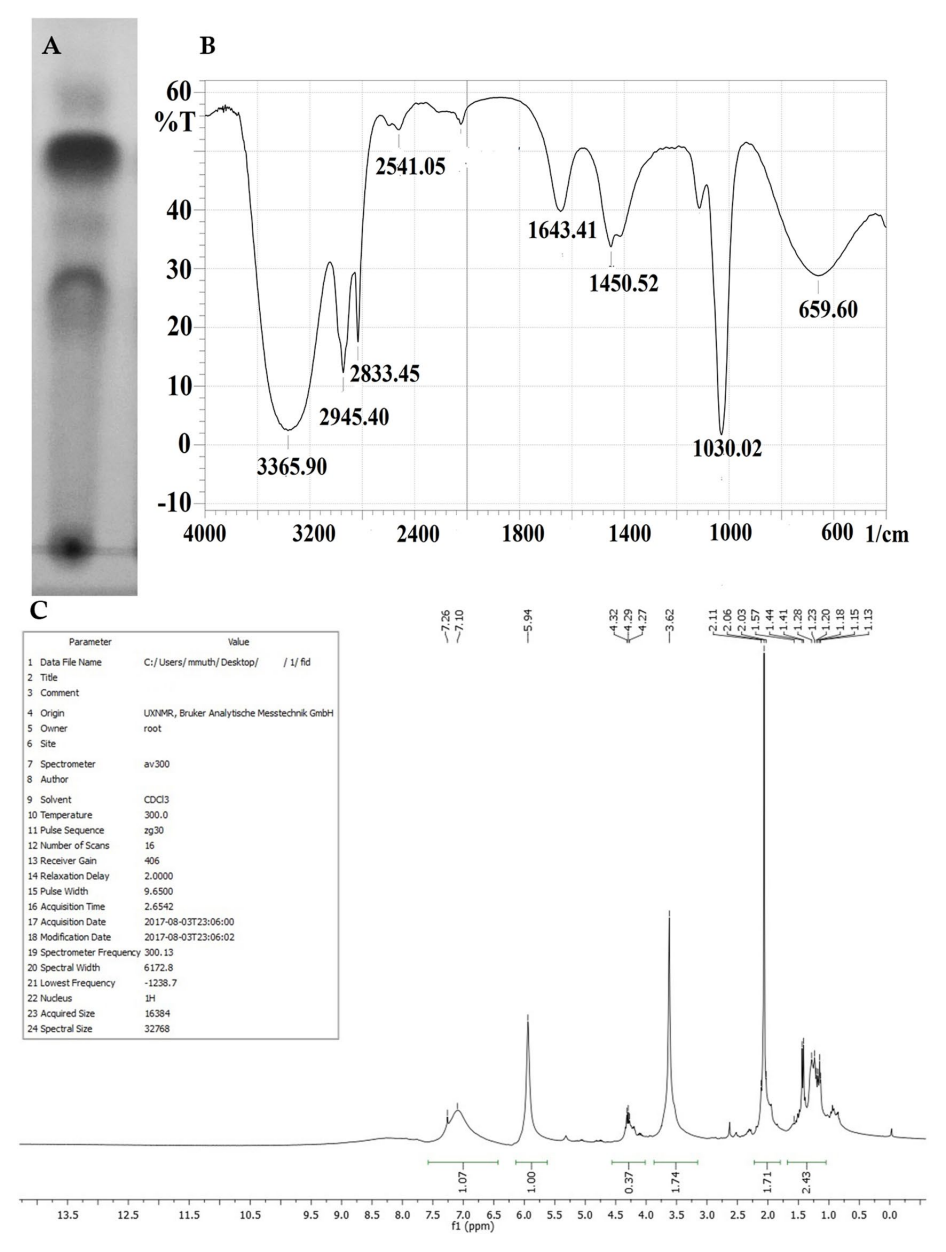
Biosurfactant characterization (**A**) TLC chromatogram of isolated biosurfactant, (**B**) FTIR spectrum of biosurfactant from *Paenibacillus thiaminolyticus* SK10. (**C**) 1H NMR spectrum for biosurfactant from *Paenibacillus thiaminolyticus* SK10.

*Paenibacillus thiaminolyticus* SK10 was grown in MRS medium for 96 h and the biosurfactant was extracted by acid precipitation. The resultant precipitant was eluted with NaCl: buffer and the highest surface-activity of the compound were observed in the fraction with 40:60 ratios of methanol and water. The surface-active fractions were collected from the column were pooled and lyophilized. The emulsification index and purity of the compound were examined. TLC plate was found to counter positive with ninhydrin reagent representing the occurrence of the peptide (Fig. 2A). The purified biosurfactant exposed the presence of lipid and peptide spots with Rf values of 0.37 and 0.53 on the silica gel TLC plates. The FT-IR spectrum results represent the existence of aliphatic groups collective with peptide moiety, a representative feature of lipopeptides (Fig. 2B). The FT-IR spectrum showed a strong broad peak at 3365.90 cm^−1^ indicates the occurrence of hydrogen-bonded –OH or –NH functional groups. Significant absorbance peak in the region of 2833 cm^−1^ and 1643 cm^−1^ corresponding to the typical acidic (COOH) group and the hydroxyl group of the amino acids. The absorbance peaks at 659, 1450, 2945 cm^−1^ shown the occurrence of an aliphatic long fatty acid chain. Additionally, the absorbance peaks at the area of 1450 cm^−1^ responsible for the presence of the aromatic carbon–carbon ring structure of the amino acid. Comparable absorption spectra in FT-IR were stated for lipopeptide in the literature. The ^1^H NMR spectrum showed the presence of a long aliphatic fatty acid chain between 1.13–1.55 ppm. Attachment of quaternary carbon with –CH_3_ moiety was confirmed with the presence of intense singlet absorbance peak at 1.57 ppm. It also exposed the presence of the CH_2_OH group of the amino acid at 4.32 and 4.27 ppm, terminal amino acid structure of COOH group, and the aromatic hydrogen atom at 7.26–7.14 ppm of an amino acid (Fig. 2C). The clear and intense signals present in the region between 5.94 showed the occurrence of amino acid Hα resonance. From TLC, FTIR, and NMR analysis, the biosurfactant produced by *Paenibacillus thiaminolyticus* SK10 was characterized as a lipopeptide.

### Synthesis of Cu-NP and its characterization

**Figure 3:**
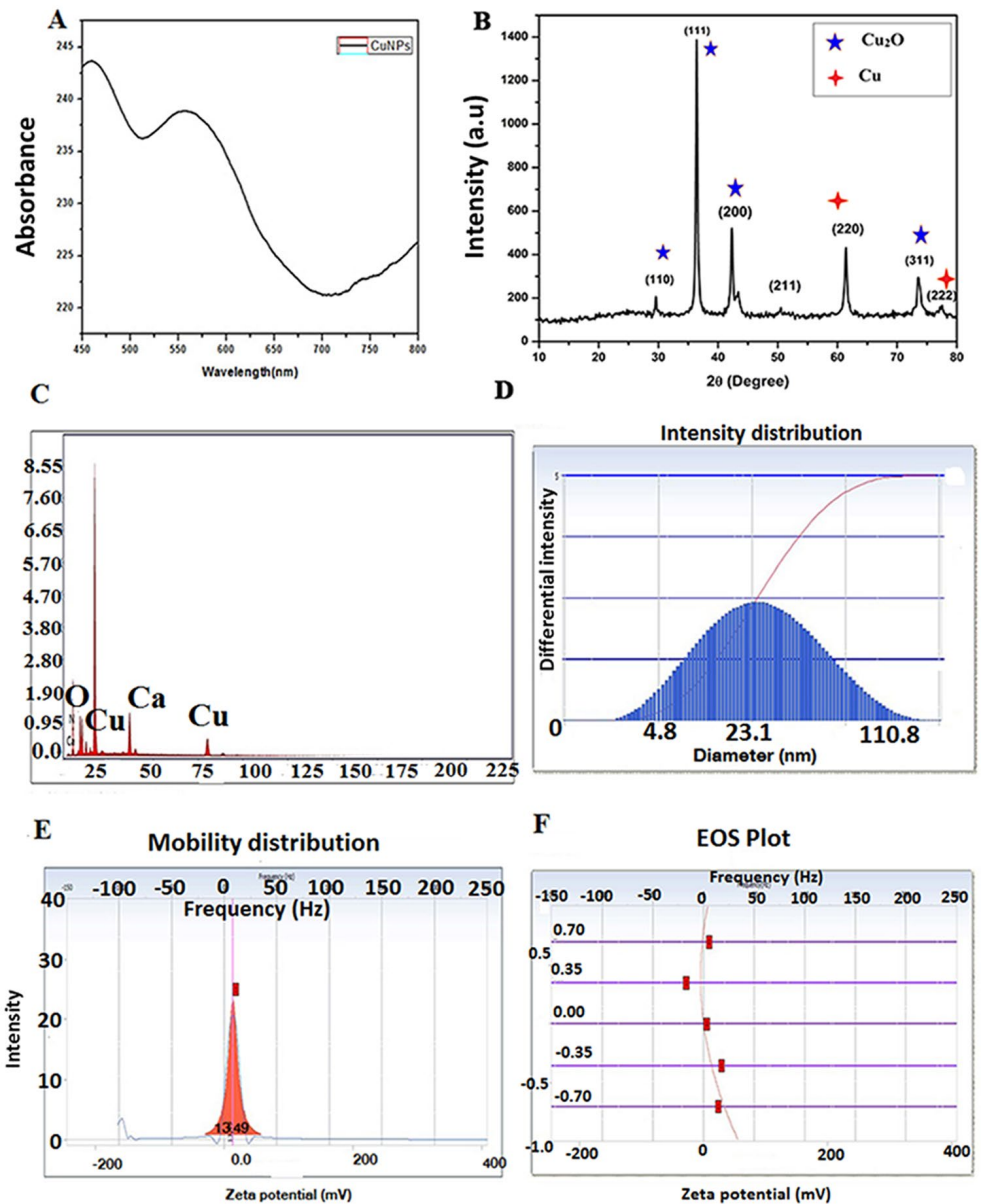
Characterization of Cu-NPs (**A**) absorption spectra of Cu-NPs obtained, (**B**) X-ray diffraction (XRD) patterns of Cu-NPs, (**C**) EDX analysis of the Cu-NPs strong signal of copper confirms the elemental composition of copper nanoparticles, (**D**) Particles size confirmed by DLS analysis, (**E**,**F**) Zeta potential measurements of Cu-NPs.

**Figure 4:**
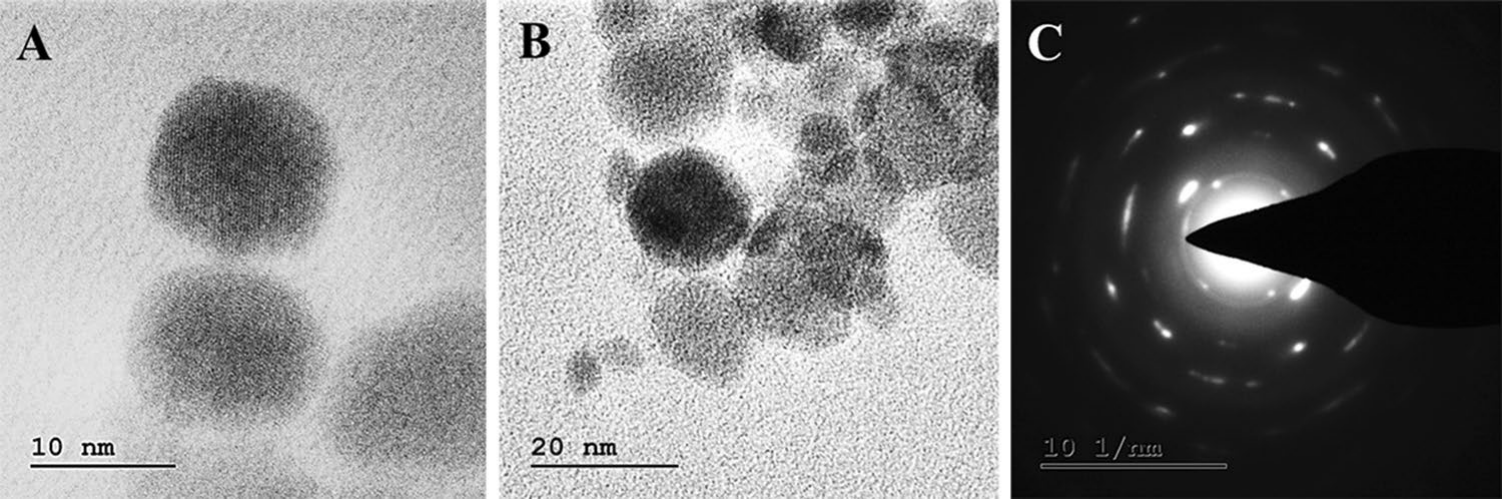
(**A**,**B**) Morphological characterization of Cu-NPs (C) SAED pattern of a single particle.

Copper nanoparticles were successfully synthesized by a chemical reduction method in water. The synthesis of the CuNPs was followed by UV–Vis analysis showed a single SPR peak at 590 nm which designates the presence of CuNPs (Fig. 3A). The crystal structure of the CuNPs was confirmed by XRD analysis. Figure 3B show the XRD patterns of the Cu and Cu_2_O nanoparticles respectively. Peaks observed at 2Ɵ values of relating to (222), (200) and (220) planes of metallic Cu. JCPDS 05–0667 equals with cubic fcc construction of Cu_2_O representing (110), (111), (200), (220), (311) and (222) planes.The composition of copper nanoparticles was analyzed by SEM–EDX (Fig. 3C). EDX spectrum designates mixer of copper and copper oxide nanoparticles with 17.43% of the copper mass. The EDX results in Fig. 3B show that the coated mixture contains other elements such as calcium (12.52%), nitrogen (1.19%), and oxygen (68.85%). The particle size results showed a mean diameter of 23.1 nm (Fig. 3D). The zeta potential of CuNPs dispersed in water was found to be 13.49 mV (Fig. 3E,F). The morphology of the CuNPs was studied using TEM with SAED mapping. Figure 4 illustrates a TEM image and the size distribution of CuNPs with a size range between 10 to 50 nm large and widely dispersed particles. Distinct diffraction rings are observed and their corresponding interplanar spacing denotes the crystalline space.

### Liposome preparation and characterization

**Figure 5:**
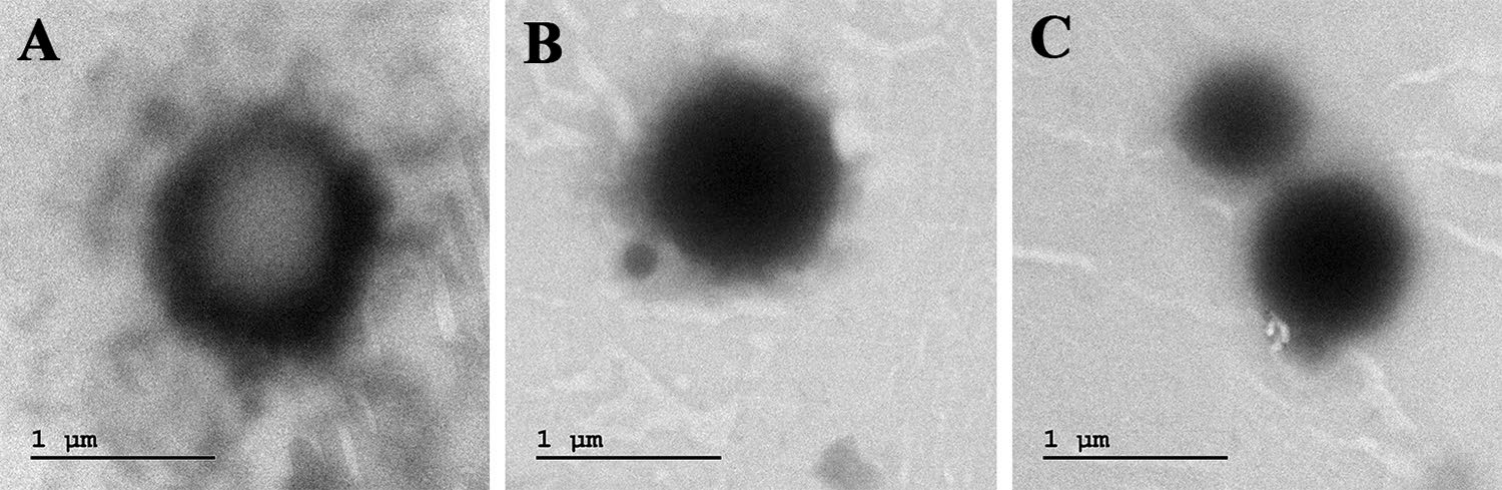
Characterization of liposomal preparation (**A**) TEM images of EL-LP-CuNPs, (**B**) Particles size confirmed by DLS analysis, (**C**) Zeta potential measurements of EL-LP-CuNPs.

Surfactant lipopeptide and CuNPs were encapsulated into the liposomes and the encapsulated liposomal characteristics were summarized below. The mean particle size of liposomes loaded with lipopeptide and CuNPs was found to be 153.4 nm. The polydispersity index (Pdi) was recorded as 0.204 is lower than 0.3 which representing the homogeneousness of the EL-LP-CuNPs respect to their size. EL-LP-CuNPs surface charge was recorded to be + 1.3 mV. The morphology of EL-LP-CuNPs was witnessed by TEM and the observation results were shown in Fig. 5. The TEM image indicates that most EL-LP-CuNPs were roughly sized spherical particles with uniform distribution. The unloaded liposomes were found to be empty and flaccid compared (Fig. 5A) with the loaded particles (Fig. 5B, C).

### Minimum inhibitory concentration (MIC)

MIC results showed no growth of *P. aeruginosa*, MRSA at the concentrations of 176 μg/ml, 223 μg/ml and above with the presence of lipopeptide. On the other hand, the MIC values for CuNPs were ranged as 197 μg/ml and 157 μg/ml for *P. aeruginosa*, MRSA respectively. In the case of EL-LP-CuNPs, the MIC value was significantly reduced up to 89 μg/ml and 105 μg/ml for *P. aeruginosa*, MRSA.

### Killing kinetics assay

**Figure 6:**
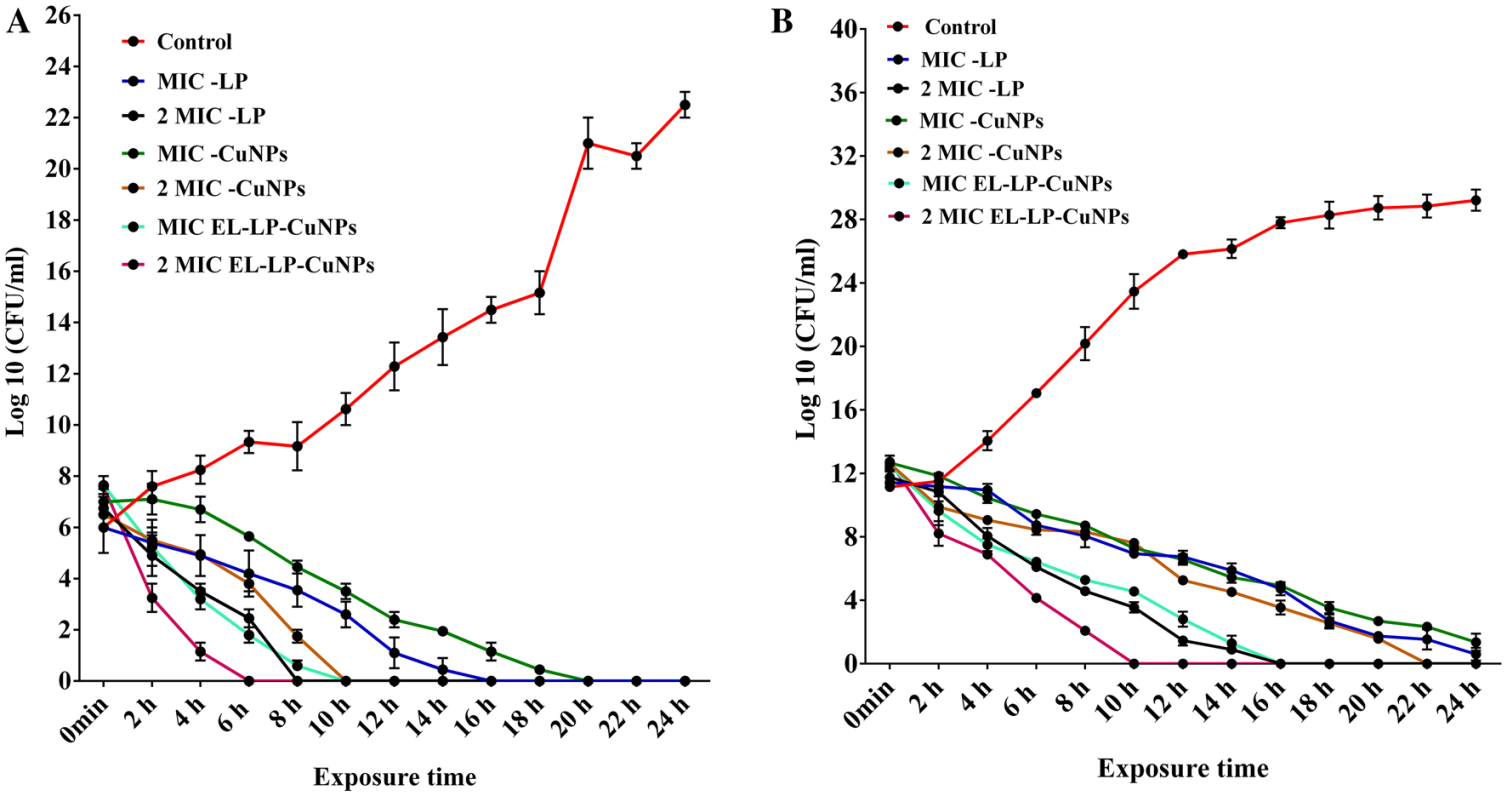
(**A**) Time-kill curves with and without the addition of drug combinations up to 24 h for *P.aeruginosa.* All data points represent the mean ± SD from at least three independent experiments. (**B**) Time-kill curves with and without the addition of drug combinations up to 24 h for MRSA. All data points represent the mean ± SD from at least three independent experiments.

During the Time-kill kinetics study, all the treatment combinations instigated declines in the number of CFU/ml over the 0 to 24 h time interval against both *P. aeruginosa*, MRSA at the MIC tested. The bactericidal activity of EL-LP-CuNPs was very fast (2 to 6 h at concentrations equal to two times the MIC) against *P. aeruginosa* and MRSA; the decrease in the total of CFU per milliliter was more than 5 log units. On the contrary, for *P. aeruginosa* and MRSA, the killing endpoint was extended up to 10 h of incubation at the MIC of LP and CuNPs treatment (Fig. 6A,B). The killing activity of LP and CuNPs was also pathogenic strain-dependent (either very fast or slow); 6 to 16 h was required for the killing of *P. aeruginosa*, but the duration was prolonged up to 24 h for the efficient killing of MRSA. The bactericidal endpoint was recorded to be 16 h (MIC LP), 8 h (2 × MIC LP), 20 h (MIC CuNPs), 10 h (2 × MIC CuNPs), 10 h (MIC of EL-LP-CuNPs), and 6 h (2 × MIC EL-LP-CuNPs) for *P. aeruginosa*. In the case of MRSA, the killing time point was 24 h (MIC LP),16 h (2 × MIC LP), 24 h (MIC CuNPs), 22 h (2 × MIC CuNPs), 16 h (MIC of EL-LP-CuNPs), and 10 h (2 × MIC EL-LP-CuNPs).

### Inhibition of carotenoid synthesis from P. aeruginosa and MRSA

**Figure 7:**
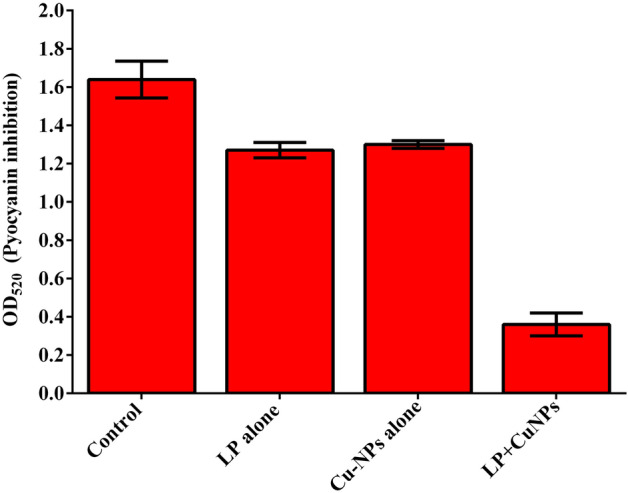
Inhibition of Pyocyanin. All data points represent the mean ± SD from at least three independent experiments.

**Figure 8:**
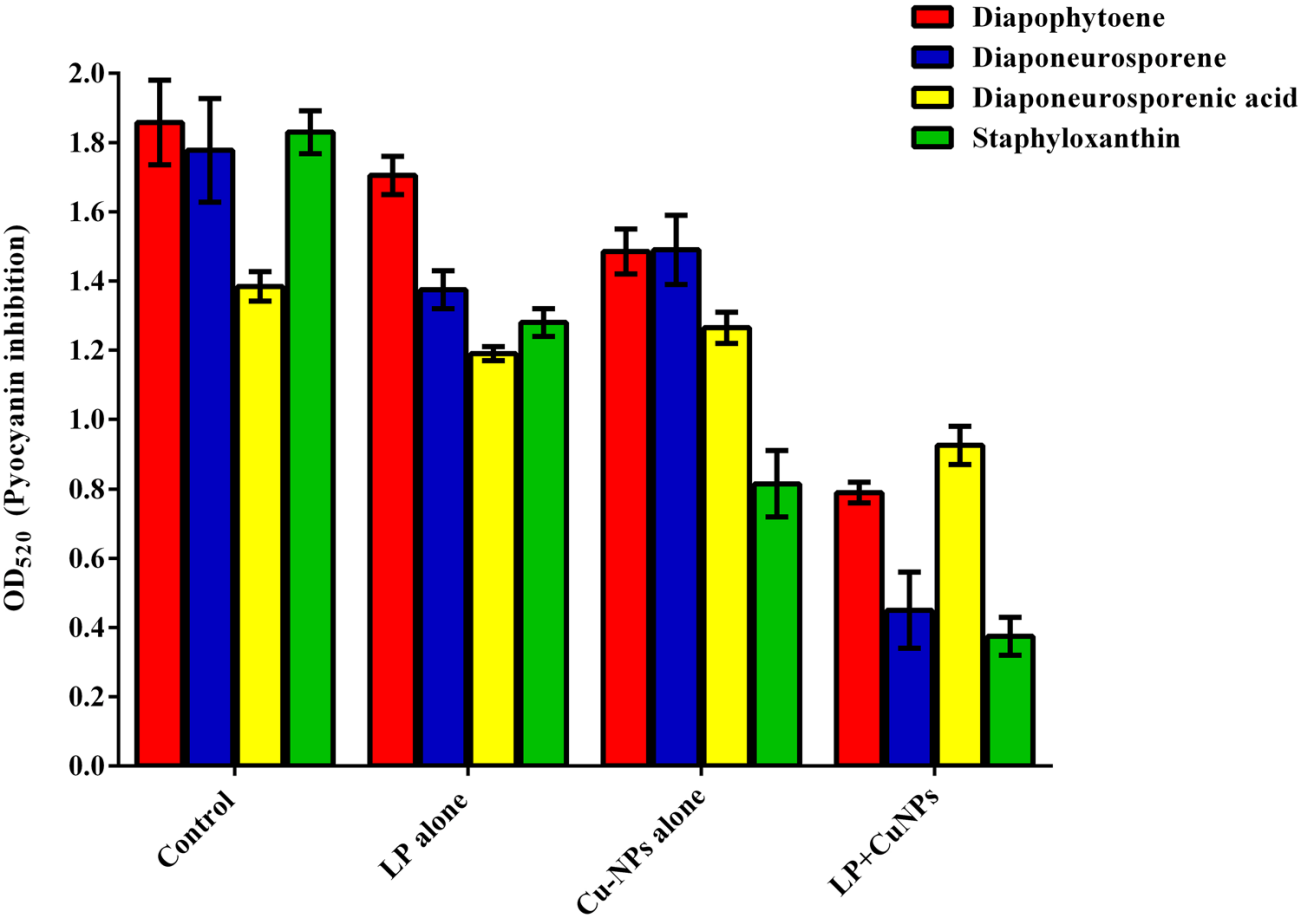
Inhibition of Staphyloxanthin. All data points represent the mean ± SD from at least three independent experiments.

The presence of LP, CuNPs, and EL-LP-CuNPs intensely decrease the capability of *P. aeruginosa* to produce pyocyanin up to 76% compared to LP alone (45%) and CuNPs alone (23%) treatments (Fig. 7). The carotenoid synthesis was assessed by measuring the amount of staphyloxanthin and its metabolic intermediates such as 4,4′-diapophytoene, 4,4′-diaponeurosporene and 4,4′-diaponeurosporenic acid and staphyloxanthin spectrophotometrically. In LP, CuNPs and EL-LP-CuNPs treated samples of MRSA the OD values were significantly reduced compared to LP alone and CuNPs alone treatment (Fig. 8).

### Inhibition of extracellular polymeric substances (EPS)

In this study, EPS secretion capability was detected from the scraped biofilms of the treated and untreated *P. aeruginosa* and MRSA. In EPS assay, EL-LP-CuNPs exhibited potential inhibition of EPS compared to LP and CuNPs. After the incubation with the 1/2 MIC of EL-LP-CuNPs, LP, CuNPs the percentage of EPS matrix decreased up to 82 ± 2%, 56 ± 4%, 42 ± 6% for *P. aeruginosa* and 77 ± 3%, 52 ± 7%, 32 ± 7% for MRSA respectively.

### Biofilm metabolic activity—XTT reduction assay

The XTT reduction assay confirmed the outcomes of MIC assay with the indication of adverse effects on the cellular viability with the treatment of all the three treatments such as EL-LP-CuNPs, LP, and CuNPs. The metabolic activity was significantly inhibited to the level of 72 and 63% in *P. aeruginosa* and MRSA with the treatment of EL-LP-CuNPs and 65% and 54% with the treatment of LP alone at ½ MIC concentrations. Likewise, the CuNPs alone inhibited the metabolic activity as 22% and 14% in *P. aeruginosa* and MRSA.

### Quantification of intracellular ROS (reactive oxygen species) production

**Figure 9:**
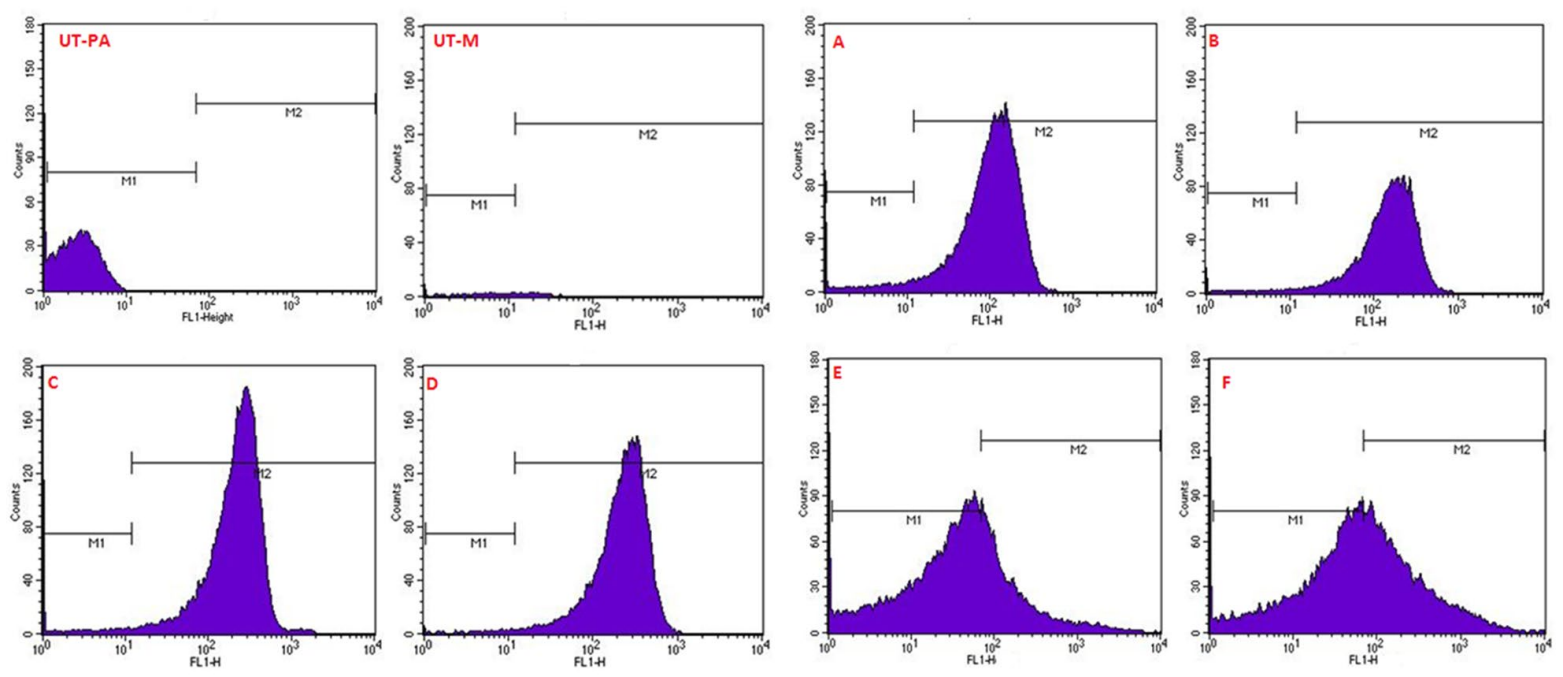
Evaluation of intracellular ROS production in *P.aeruginosa* and MRSA cells following treatment with LP, Cu-NPs and LP + Cu-NPs. The concentration selected for the study was based on their respective MIC values. H_2_O_2_ treated cells were used as positive control while cells alone were used as negative control.

To confirm the changes in the generation of ROS during the treatment of EL-LP-CuNPs, LP, and CuNPs, flow cytometry with H_2_DCFDA staining was performed. Cytometric findings showed that intracellular ROS concentrations are considerably increased by EL-LP-CuNPs, LPs, and CuNPs, suggesting that a combination of treatment outcomes in the accumulation of ROS. Intracellular ROS was found to be slightly elevated in the CuNPs exposed cells. Relative ROS production was significantly higher in the EL-LP-CuNPs treated groups than in the LP, and CuNPs groups in both bacteria (Fig. 9).

### Confirmation of membrane damage and cell death through flow cytometry

**Figure 10:**
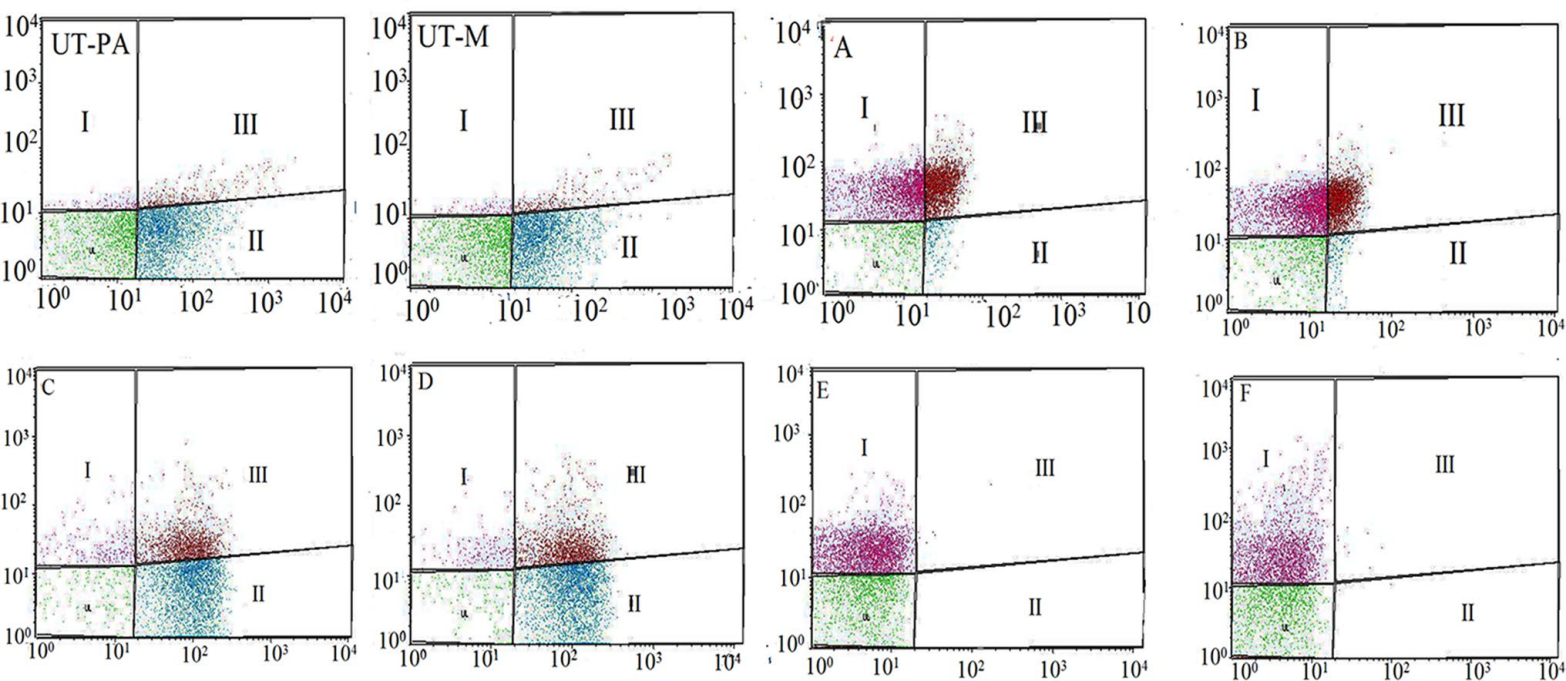
Membrane integrity assessment by live/dead staining of *P. aeruginosa* and MRSA cells by flow cytometer. Flow cytometer scatters plot for (UT-PA) untreated *P. aeruginosa* cells, (UT-M) untreated MRSA cells (**A**) LP treated *P. aeruginosa* cells stained with SYTO9/PI stains were analyzed, (**B**) LP treated *MRSA* cells stained with SYTO9/PI stain, (**C**) Cu-NPs treated *P. aeruginosa* cells stained with SYTO9/PI, (**D**) Cu-NPs treated *MRSA* cells stained with SYTO9/PI, (**E**) EL-LP-CuNPs treated *P. aeruginosa* cells stained with SYTO9/PI, (**F**) EL-LP-CuNPs treated *MRSA* cells.

Membrane damage was confirmed by the uptake of PI by treated cells. Figure 10. shows the results obtained when MRSA and *P. aeruginosa* were exposed to LP, CuNPs, and EL-LP-CuNPs for 4 h. In the dot plot results, the control samples represent the bacterial population without drug exposure. The green dots show the proportion of ‘live’ cells whilst the red and pink dots symbolize the proportion of ‘dead’ cells in suspension after incubation. From these results, it can be concluded that EL-LP-CuNPs treatment exhibit significant antibacterial activity against *P. aeruginosa* and MRSA as a large proportion of the cells were injured (~ 88% ± 0.18). However, a strong antimicrobial effect was observed LP (> 59% dead) and CuNPs with most cells showing damaged membranes (> 48%).

## Biofilm inhibition assays

### Assessment of biofilm biomass

**Figure 11:**
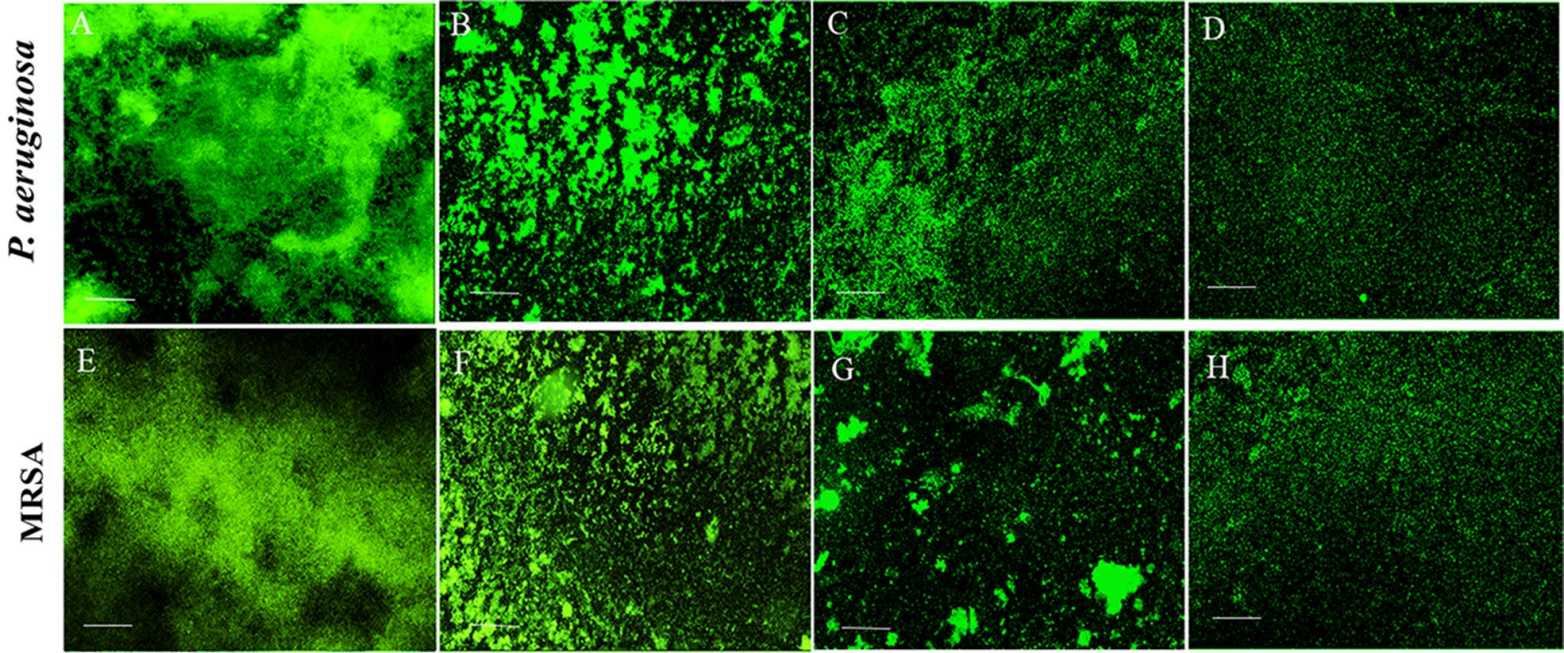
Acridine orange stained *P.aeruginosa* and MRSA biofilms with treatment combinations. (**A**,**E**) Control biofilms without treatment, (**B**,**F**) Biofilms treated with LP alone, (**C**,**G**) Biofilms treated with Cu-NPs alone, (**D**,**H**) Biofilms treated with LP + Cu-NPs (50 µM).

Both the LP alone and CuNPs alone presented rational biofilm inhibition ability in the order of 65%, 45% for *P. aeruginosa,* and 59%, 47% for MRSA*.* The experiment with EL-LP-CuNPs treated samples showed nearly 85% and 82% reduction of preformed biofilms in *P. aeruginosa* and MRSA. These effects designate that the EL-LP-CuNPs is an effective antibiofilm solution for both *P. aeruginosa* and MRSA biofilms (Fig. 11).

### High content screening (HCS) imaging of biofilm

**Figure 12:**
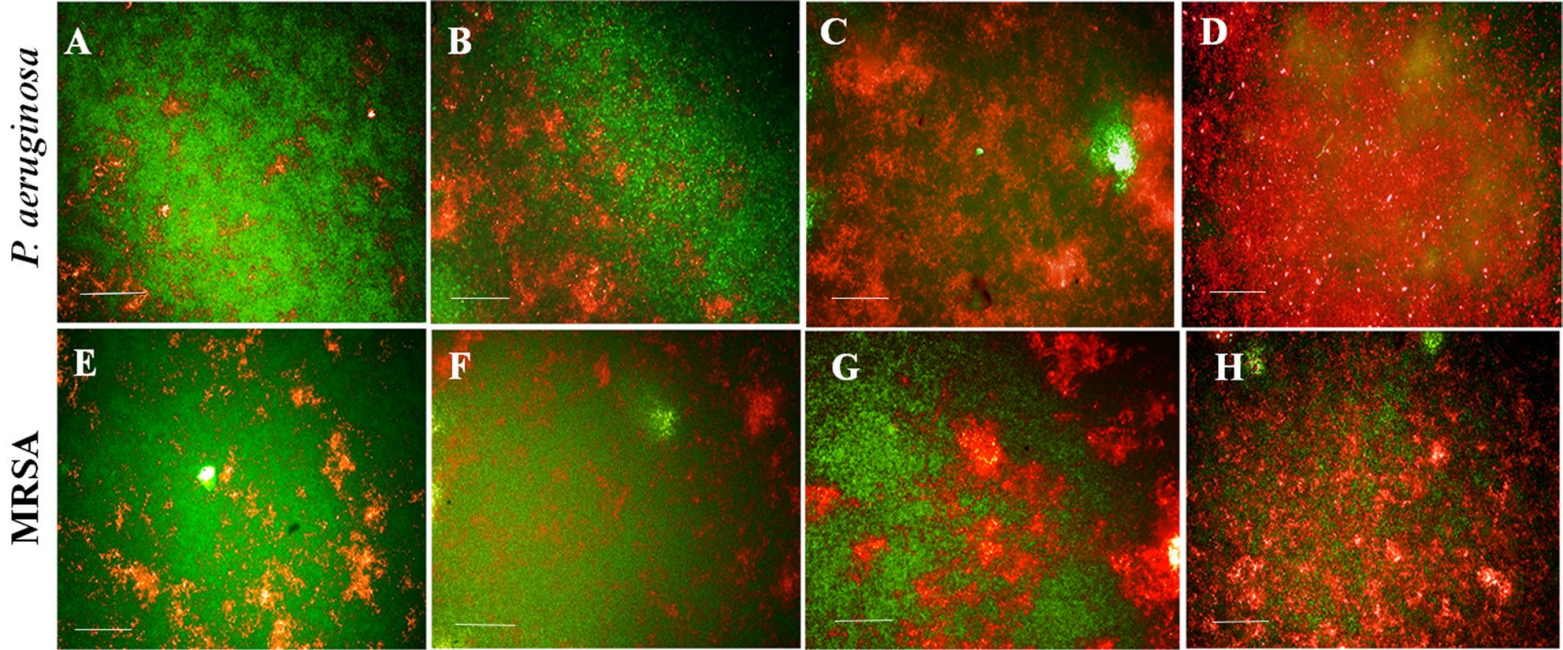
Membrane integrity assessment of preformed biofilms—Live and dead cells differentiated syto 9 & PI stained *P.aeruginosa* and MRSA biofilms with treatment combinations. (**A**,**E**) Control biofilms without treatment, (**B**,**F**) Biofilms treated with LP alone, (**C**,**G**) Biofilms treated with Cu-NPs alone, (**D**,**H**) Biofilms treated with LP + Cu-NPs (50 µM).

The high content fluorescent imaging analysis revealed that the control biofilms were appressed and firm with multiple layers. Conversely, the treated biofilms were disorganized and detached from the wells. During the EL-LP-CuNPs treatment, the thickness and adherence of the film were reduced. The film has consisted of sparsely scattered very small aggregates of cells (Fig. 11). The biofilms treated with LP alone and CuNPs alone were less extensive than the untreated control. Using syto9/PI, both live (green) biofilm embedded cells and dead (red) cells were observed (Fig. 12). HCS imaging with live and dead staining exhibited strong bactericidal activity against both *P. aeruginosa* and MRSA with the treatment of ½ MIC concentrations. HCS images of EL-LP-CuNPs treatment displayed an increased PI intensity and inferior intensity of green fluorophore due to syto 9 which discernibly validated the prodigious killing efficiency (Fig. 12).

### Scanning electron microscopic analysis of biofilm

**Figure 13:**
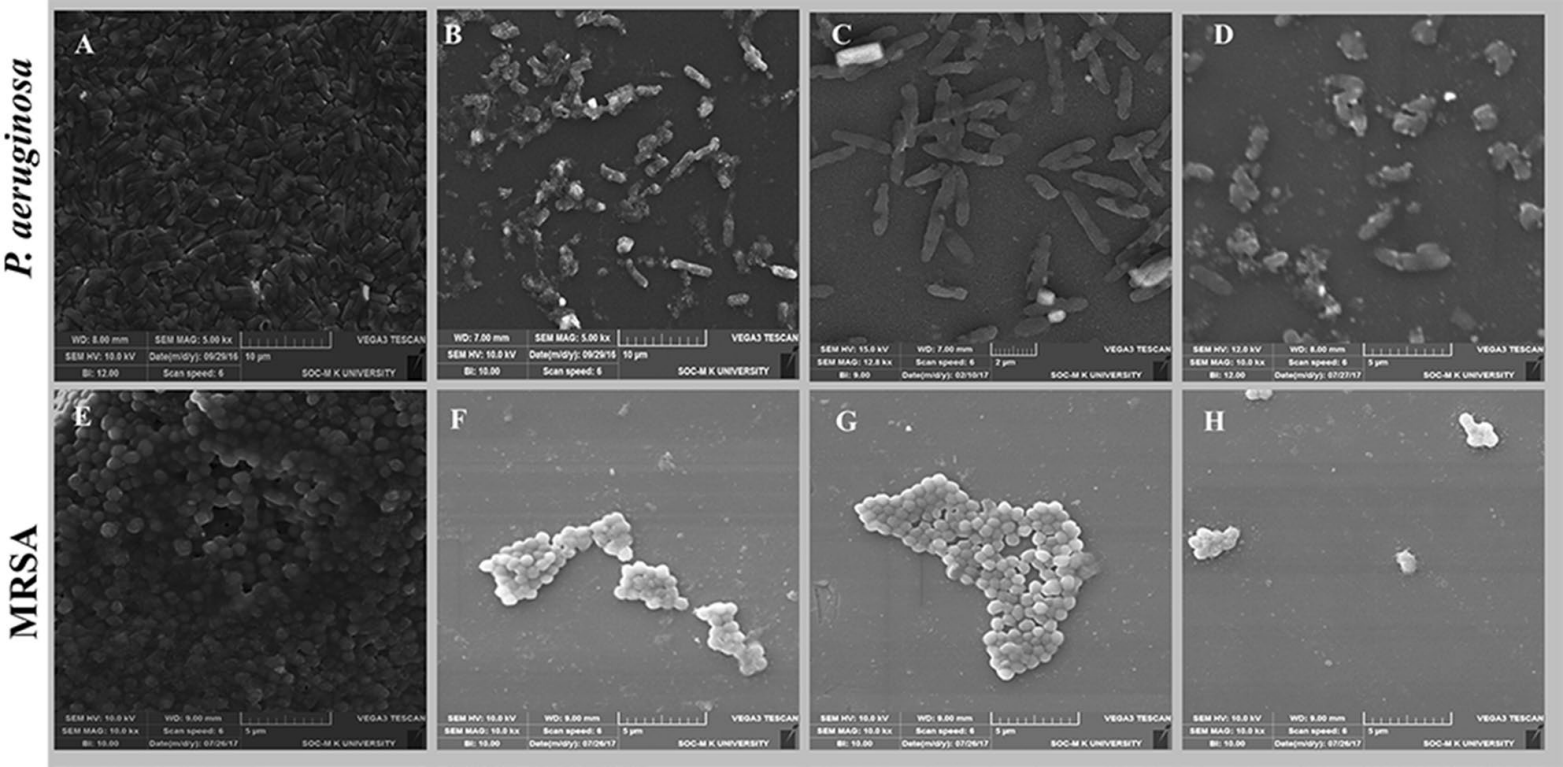
SEM images of *P.aeruginosa* and MRSA biofilms with treatment combinations. (**A**,**E**) Control biofilms without treatment, (**B**,**F**) Biofilms treated with LP alone, (**C**,**G**) Biofilms treated with Cu-NPs alone, (**D**,**H**) Biofilms treated with LP + Cu-NPs.

SEM analysis was performed to compare the biofilm morphology of treated and untreated cells. Both the untreated *P. aeruginosa* and MRSA biofilms were composed of accumulated rods and cocci with the extracellular matrix-like film (Fig. 13A,E). Biofilms treated with lipopeptide alone showed disrupted films comprises scattered microcolonies surrounded by a thin extracellular polymeric matrix (Fig. 13B,F). Whereas cells treated with CuNPs appeared elongated with membrane ruptures in *P.aeruginosa* and MRSA (Fig. 13C,G). The augmented antibiofilm effect was noticed with the presence of EL-LP-CuNPs due to the synergistic action of LP with CuNPs.With the treatment of EL-LP-CuNPs, almost all the cells were detached from the biofilm. Completely disrupted films with damaged cells were observed in EL-LP-CuNPs treatment (Fig. 13D,H).

### Bacterial ultra-structural analysis by TEM

**Figure 14:**
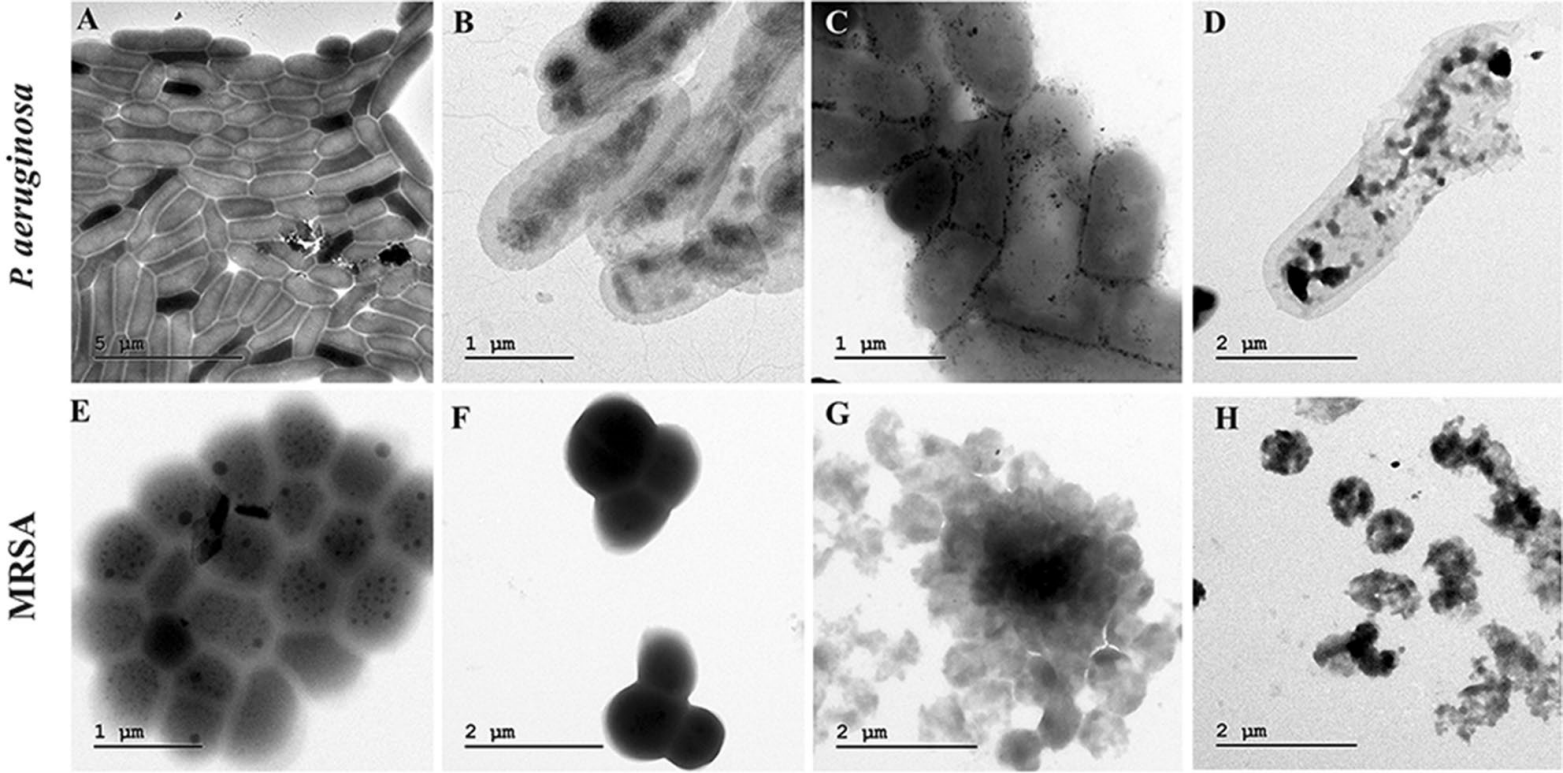
TEM Ultrastructural morphological damages of *P.aeruginosa* and MRSA biofilms with treatment combinations. (**A**,**E**) Control biofilms without treatment, (**B**,**F**) Biofilms treated with LP alone, (**C**,**G**) Biofilms treated with Cu-NPs alone, (**D**,**H**) Biofilms treated with LP + CuNPs.

The transmission electron micrographs exhibited a significant change in bacterial cell structure after exposure (Fig. 14). For *P. aeruginosa* and MRSA, control cells showed an even and dense superficial morphology, without leakage of intracellular constituents and absence breaks or pores on the surface of the cell. In divergence, treated bacterial cells showed an extensive range of substantial aberrations on the cells. When bacterial suspensions were exposed to CuNPs alone, there were some CuNPs were adhering to the surface of both *P. aeruginosa* and MRSA cells. A profound collapse of the cell arrangement was found with the treatment of LP was observed. Besides, there was severe destruction on the cell walls and a superficial hole at cell poles with loss of intracellular contents was observed with the treatment of EL-LP-CuNPs. Lysed cells were enclosed by shady floccules.

### Coating of urinary catheters with EL-LP-CuNPs for the in vitro antibiofilm activity

**Figure 15:**
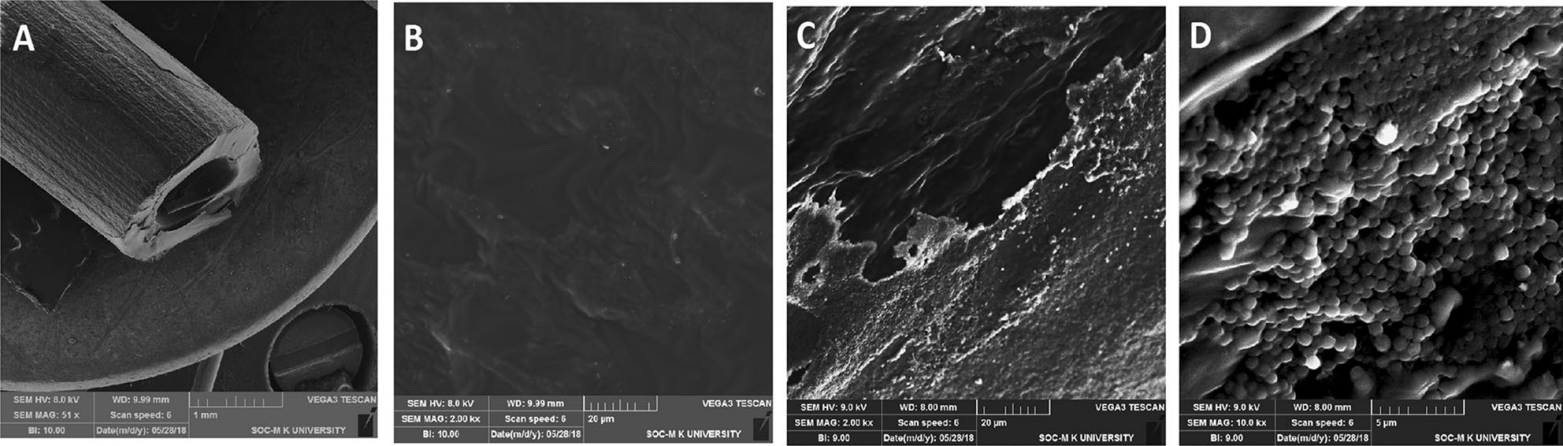
Representative SEM images of dense biofilm formation on the surfaces of uncoated (**A**,**C**,**D**), EL-LP-CuNPs coated catheters (**B**).

A noteworthy variance between uncoated and EL-LP-CuNPs coated catheters was perceived about the mean number of viable colonies on colonized catheters. Figure 15 shows that uncoated catheters which are preincubated in PBS and exposed to 10^6^, 10^8^, CFU/ml of *P.aeruginosa,* and MRSA, respectively, were occupied comprehensively with the pathogens, while EL-LP-CuNPs coated catheters were devoid of bacteria (Fig. 15). In the case of uncoated catheters, the total of adherent *P. aeruginosa* and MRSA ranged from 2.7 ± 0.5 and 5.3 ± 0.7 log10 CFU/catheter. When exposed to 10^6^ and 10^8^ CFU/ml of *P. aeruginosa* and MRSA, only 8 and 17 colonies were observed in the EL-LP-CuNPs coated catheters. The exteriors of uncoated and EL-LP-CuNPs coated catheters were observed by SEM after colonization with *P.aeruginosa* and MRSA. SEM on EL-LP-CuNPs coated catheters presented bacteria entrenched inside a more multifaceted matrix covering than that of uncoated catheters. The uncoated catheters were devoid of bacterial adherence and covered with a homogenous layer of EL-LP-CuNPs.

## Evaluation of in vivo therapeutic potential on C. elegans

### Survival and in-vivo adherence using C. elegans

**Figure 16:**
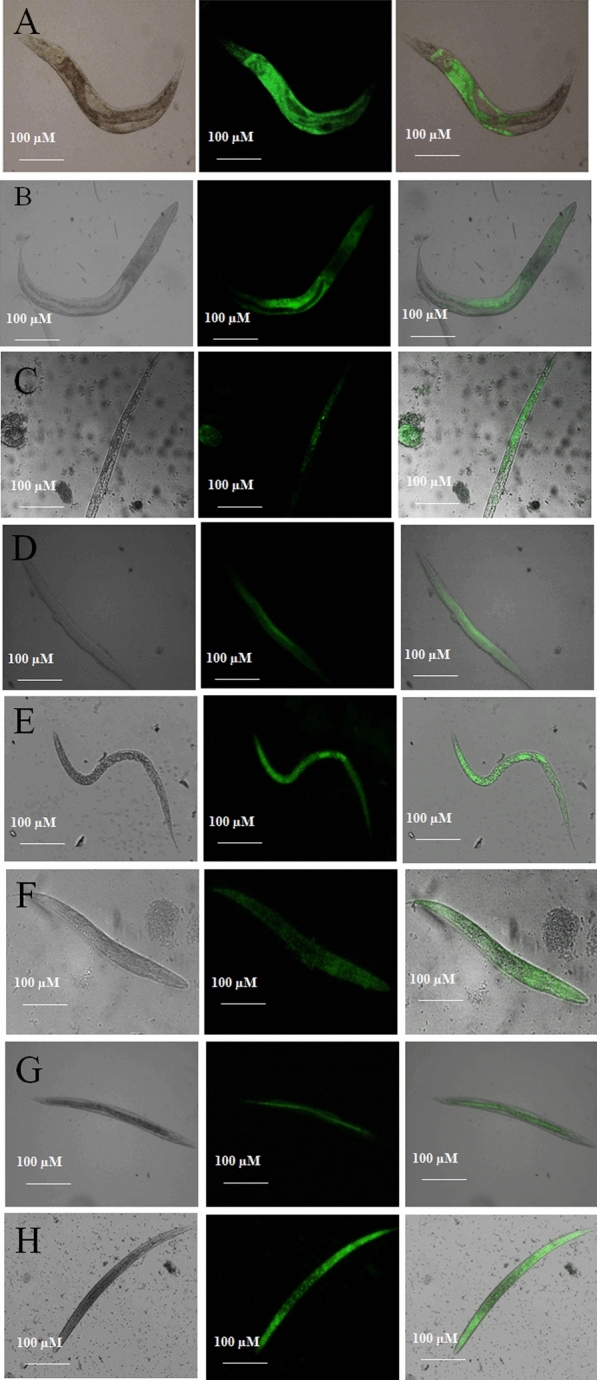
HCS images of colonization by *P.aeruginosa* and MRSA under treatment conditions. (**A**) *C. elegans* infected with *P.aeruginosa,* (**B**) *C. elegans* infected with P.aeruginosa and treated with LP alone, (**C**) *C. elegans* infected with *P.aeruginosa* and treated with Cu-NPs alone, (**D**) *C. elegans* infected with *P.aeruginosa* and treated with LP + Cu-NPs, (**E**) *C. elegans* infected with MRSA, (**B**) *C. elegans* infected with MRSA and treated with LP alone, (**C**) *C. elegans* infected with MRSA and treated with Cu-NPs alone, (**D**) *C. elegans* infected with MRSA and treated with LP + Cu-NPs.

Significant variances in survival between worms fed with *E.coli* OP50 and virulent *P. aeruginosa* and MRSA were seen. The mean survival rate was extended by EL-LP-CuNPs up to 68.75%. LP alone and CuNPs alone increased mean lifespan by 52%, 51.42% respectively with the infection of *P. aeruginosa* (Fig. 16). The adherence assay was conducted on different treatment conditions, worms were analyzed for colonization by *P. aeruginosa* and MRSA. It was found that untreated *P. aeruginosa* and MRSA had the ability to colonize the gut of *C. elegans*. The bacterial burden inside the *C. elegans* was designated by the fluorescence intensity in the nematodes (Fig. 16). As anticipated, the untreated worms colonized by *P. aeruginosa* and MRSA displayed more strong fluorescence compared to the treated groups. Very weak colonization with less fluorescence was observed in EL-LP-CuNPs treated groups.

### Evaluation of colonization by CFU assay

**Figure 17:**
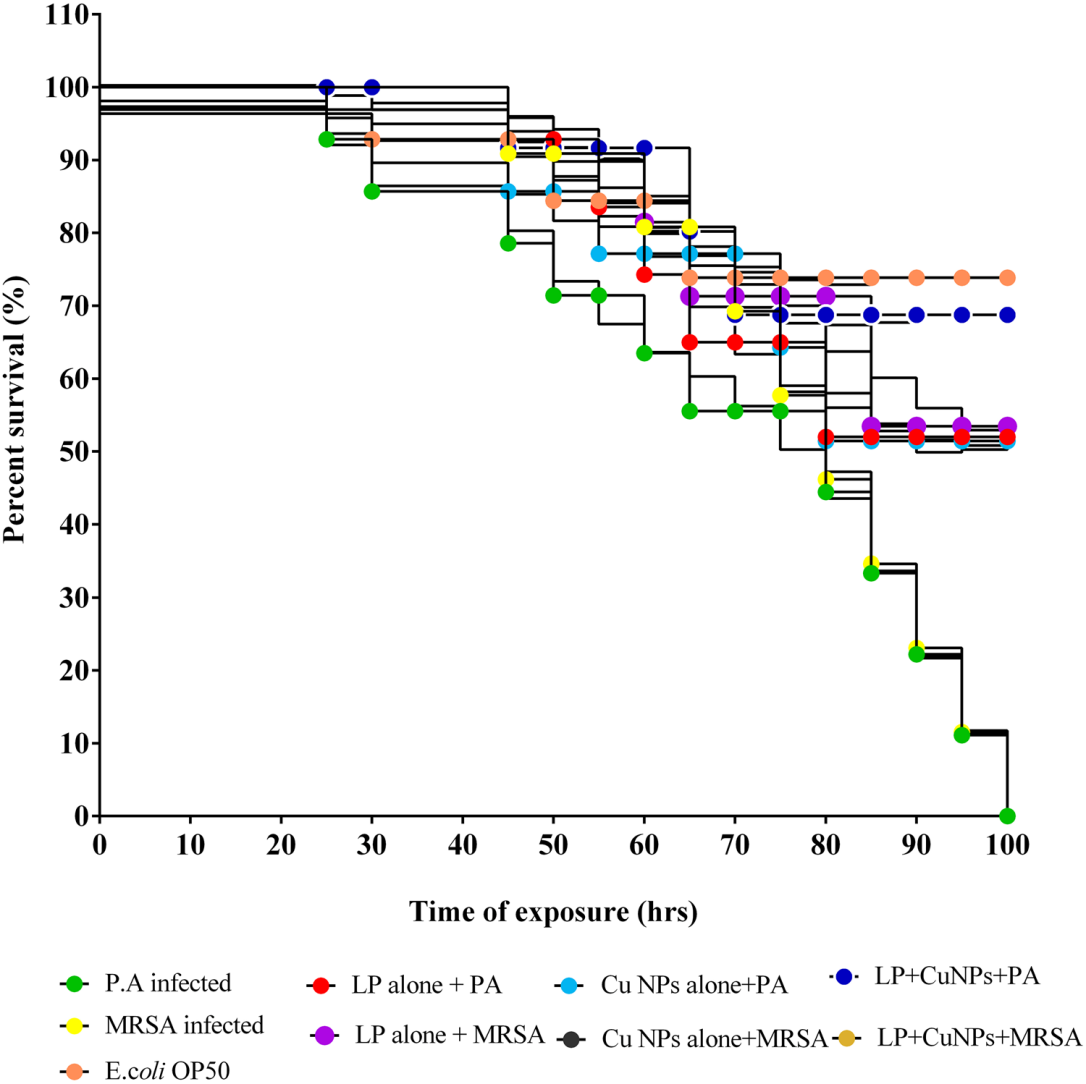
Kaplane-Meier survival plots from *C. elegans* feeding on *P. aeruginosa*, MRSA upon the treatment of LP alone, Cu-NPs alone, LP + Cu-NPs. All data points represent the mean ± SD from at least three independent experiments.

To further determine the results of adherence, the CFU assay was performed. The CFU of 4 h *P. aeruginosa* exposed nematode was 5.7 × 10^6^ CFU/mL and it was found to be declined to 4.3 × 10^5^, 3.8 × 10^5^ and 2.1 × 10^3^ CFU/mL with the treatment of LP alone, CuNPs and EL-LP-CuNPs respectively (Fig. 17).

## Discussion

The main problem of biofilm-based infections is resistance to antimicrobial agents which lead to the eventual development of multidrug resistance. Subsequently, it develops challenges to treat the infection by bactericidal treatments through systemic administration and demands the requirement for the improvement of novel antimicrobial and anti-biofilm drugs^12^. As a substitute to available antibiotic treatment and to manage the infections, we hereby report a combination therapy as potential drug candidates in the light of our earlier studies. In general, tolerance to acidic conditions and cathelicidin peptides has been measured as a favorable situation for the establishment and metabolic activity of bacteria in human skin^13^. Hence, being in the media containing acid and human cathelicidin LL-37, the promising isolate, *Paenibacillus thiaminolyticus* SK10 was explored for the production and characterization of biosurfactant. The surface-active antibacterial fraction was identified as a lipopeptide based on TLC analysis and further confirmed by IR spectra and NMR spectra. Comparable to the current study, linear lipopeptide surfactants have been stated previously from marine bacteria, such as Pontifactin, Rhodofactin, and Brevifactin^14^. Lipopeptide with antimicrobial potential established to date is synthesized by microorganisms of terrestrial origin. Very limited reports only stated on antibacterial lipopeptide molecules from human skin microflora.

Although the antimicrobial activity of lipopeptides has been formerly stated, little is reported about their interaction with nanoparticles for the enhanced antibacterial activity. UV–visible spectral study showed that the CuNPs presented surface plasmon resonance at 540 nm. The establishment of non-oxidized CuNPs at 580 nm was due to strong surface plasmon resonance and exhibits plasmon resonance at 556–580 nm in general. Zeta potential is measured as a degree of charges on the surface of the nanoparticles, the greater magnitude of which progresses dispersal strength of nanoparticles. The zeta potential of CuNPs dispersed in water was found to be 13.49 mV at a pH of 6.8 which may be recognized to the formation of hydroxyl groups. Zeta potential of the nanoparticle can significantly influence its stability in suspension through the electrostatic repulsion among particles. It can also regulate the in vivo interaction of the nanoparticle with the cell membrane of bacteria. When the anti-microbial particle is positively charged, this may decrease the chance of bacterial adhesion which eventually influences biofilm formation^15^. The EDX peak positions were consistent with the Copper nanoparticle and sharp peaks of EDX designate the crystalline nature. The sizes of the CuNPs were further confirmed with XRD calculated from the Debye–Scherrer equation to be about 22 nm. The XRD spectra were reliable with the metallic copper and the consistent peak position was also noted and compared with previous reports^16,17^. Additional structure and morphology of the CuNPs were confirmed by TEM analysis. The result of the SAED pattern is in near covenant with the XRD results. The diffraction peaks of XRD profile and clear circular fringes of SAED pattern represent the high crystalline nature of biosynthesized CuNPs. The concern of the bactericidal activity of copper metal ions released from nanoparticles has been the topic of substantial discussion in recent years. The principle agent seems to be the ionic species, important in the electrostatic attraction among negative or positively charged cell membrane of the microorganism. The growth of both *P. aeruginosa*, MRSA was repressed by the CuNPs, with the MIC of 197 μg/ml and 157 μg/ml respectively. The resulted MIC values of CuNPs were consistent with the values of chitosan nanoparticle–loaded copper ions previously stated by Ma et al*.*^18^.

Studies representing that antimicrobial peptides act more efficiently in combination with other antimicrobial metallic nanoparticles. The results of the synergistic action of CuNPs with lipopeptide validated the potential of CuNPs to augment antibacterial action and, therefore, support previous findings on synergistic action between nanoparticles and antibiotics^19^. Drug carriers can provide the means by several mechanisms to overcome restrictions of combining two different drugs to improve its activity and thus contribute to the therapeutic efficacy. Antimicrobial liposome encapsulation possibly provides improved pharmacokinetics, pharmacodynamics, and reduced toxicity compared to conventional formulations. In the present work, we have devised liposome encapsulation of lipopeptides with CuNPs for antimicrobial therapy to address specific therapeutic efficacy and antibiofilm potential. It has been demonstrated that the application of drug-containing liposomes not only improves the cytoplasmic delivery of liposome-containing compounds but also has its bactericidal attributes. A number of fold increase in antivirulent potential was detected in EL-LP-CuNPs than individual lipopeptide and CuNPs, this may be due to the fusion of liposomes with the outer membrane of pathogen thereby releasing the drug. Also, time-killing kinetics studies of drug combinations direct a good killing effect for *P.aeruginosa* 16 h; the effect against MRSA was extended up to 24 h. These outcomes are in agreement with the results of 24-h killing curve studies described formerly. Reliable with earlier studies, LP, CuNPs, and EL-LP-CuNPs demonstrated a range of activity against both *P.aeruginosa* and MRSA.

MRSA synthesize a carotenoid pigment staphyloxanthin, for its persistence during the existence of oxidants. Inhibition of staphyloxanthin makes the MRSA cells sensitive to antimicrobial agents and the immune system. The measurable pigment was expressively inhibited with all the treatment conditions. Previously Leejae et al. reported that the rhodomyrtone mediated inhibition of staphyloxanthin with increased production of 4,4ʹdiapophytoene^20^. In *P. aeruginosa* redox-active pyocyanin was considered as an important virulence factor excreted at high cell density through the influence of Las and Rhl AHL signal molecules. The pigment pyocyanin provides green color to *P. aeruginosa* which can be accessibly observed by UV/Vis absorbance. Treatment with CuNPs lipopeptides intensely declines the ability of *P. aeruginosa* to produce pyocyanin. It is evident from our study that the CuNPs with LP combination showed 54 and 47% inhibition of the EPS production in *P.aeruginosa* and MRSA. To date, no lipopeptide and nanoparticle combination is stated to constrain the synthesis of EPS which is very vital for biofilm development. Henceforward, inhibition of EPS production will prompt the infectious biofilm exposure to the antimicrobial agent and that in turn will assist the suppression of biofilm.

One criterion for distinguishing between viable and dead bacterial cells is the integrity of the cell membrane. It is claimed that viable cells have intact and tight cell membranes that cannot be accessed by certain staining compounds, while dead cells are deemed to have damaged and/or broken membranes. Syto 9 (Green fluorescent is a cell membrane-permeable agent stains only live cells) PI (impermeable reagent stains only the damaged cell’s nuclei) fluorescence intensity was measured with flow cytometry. Histogram Data in Fig. 12. show that membrane permeability was changed after LP and CuNPs treatment and penetrability improved with EL-LP-CuNPs exposure. Discharge of ROS in bacterial cells has been studied to have an impact on survival. Flow cytometry ROS detection showed that EL-LP-CuNPs augment ROS generation in *P.aeruginosa* and MRSA cells by 63% and 75% respectively. Therefore, we consider that the antimicrobial ability of the EL-LP-CuNPs involves the generation of intracellular ROS. Raise of ROS levels is the key candidate intermediaries for cell death. ROS production could be triggered by impeded electronic transport in the damaged plasma membrane along the respiratory tory chain^21^.

The free-floating cells amassed to form biofilm assembly with the assistance of several features viz., initial attachment, motility, cell proliferation, and accumulation of multilayer bacterial masses, which finally lead to the development of a multifarious polymeric matrix. Therefore, control of bacterial biofilm could substantively weaken disease related to virulence factors^22,23^. In the present study, the results of the HCS analysis shown that the architecture of the lipopeptide treated biofilms was unattached and scattered compared with that of control biofilms. This might be due to the collective influence of LP and CuNPs where NPs acts as antibacterial agents lessening total bacterial population into the suspension and LP were altering the physicochemical properties of the surface hence restraining bacterial cell attachment from the surface. This result validates that the EL-LP-CuNPs showed 3–4 folds superior anti-biofilm activity even at the lowest dose as compared to the previous reports where 7.5 mg/ml of rhamnolipid was used.

Membrane integrity was deprived of the exposure to CuNPs alone which agrees with the findings of Sivaranjani et al.^24^. Dense and mature biofilms are tremendously tough to most of the antibiotics^25^. Here in the present study, the biofilms where disrupted efficiently with the help of LP alone which makes the cells susceptible to CuNPs for the membrane disruptions. The present experiment, evidenced has that combinatorial EL-LP-CuNPs was considered to be a trustworthy antimicrobial and antibiofilm agent proficient of efficaciously preventing pathogenic biofilm of medical concern.

Furthermore, in SEM and TEM results, we found that Gram-positive MRSA was more damaged with EL-LP-CuNPs combination in comparison to Gram-negative *P.aeroginosa* despite the existence of the profuse peptidoglycan layer. It has been accepted in the literature that Gram-positive bacteria cell wall teichoic acid is commonly the binding site of some small molecules and nanoparticles^26^. The augmented vulnerability of MRSA may be attributed to the interaction of EL-LP-CuNPs with the anionic teichoic acid which leads to the cleavage of the peptidoglycan layer and formation of pores. When these pores are formed in the pathogen cell membrane, the quantity of antimicrobial drug receiving entree to bacterial cytosol may be boosted and the cell would become more susceptible to antibiotics. The observation of the flow cytometry and TEM results has provided support to the mechanism. The augmented cell death witnessed in the present study hints upon encapsulation of LP and CuNPs in liposomes which have caused the enhanced cell membrane damage and lead to the elevated release of reactive oxygen species (ROS).

A greater percentage of catheter-mediated infections are caused by pathogens such as *P*. *aeruginosa,* *S. aureus*, *Enterococcus,* and *E. coli*. To effectively examine the EL-LP-CuNPs facilitated alterations on the surface topology and architecture of biofilms formed on the surface of the catheters, and in situ microscopic examination was performed using SEM. From our results, it has been clear that catheters coated with EL-LP-CuNPs are very operative in inhibiting the development of biofilm formation and growth of *P.aeruginosa* and MRSA in vitro. In this context, EL-LP-CuNPs catheters should be considered as one more weapon in the fight against infection to subside pathogen implantation, to slow their consequent growth and to impede biofilm development.

*P.aeruginosa* kills the host *C. elegans* with the help of cyanides and quorum-sensing controlled-virulence factors. MRSA infects the worms through the synthesis of pore-forming cytolysins, neuromuscular endotoxin, and proteases such as gelatinase and serine protease. Survival and *in-vivo* adherence, and CFU assay determined the anti-infective and *C. elegans* protection efficacy of EL-LP-CuNPs against LM infection. The results of the present examination and the strongly suggest that EL-LP-CuNPs preparation is a favorable broad-spectrum anti-virulence therapeutic agent for the treatment of bacterial infections.

## Materials and methods

All the methods were carried out in accordance with relevant guidelines and regulations.

### Pathogens used and their culture conditions

*P. aeruginosa* ATCC 25,619 and MRSA ATCC 43,300 are the target bacterial pathogens used in the present study. *P. aeruginosa* and MRSA were cultivated at 37 °C for 24–48 h, maintained, and assays were performed in King's B broth (KBB; Himedia, India) (pH 7.0) and tryptone soy glucose broth (TSBG) respectively.

### Consent to participate and ethical approvals

Study participants provided informed written consent to participate in the study.Research was implemented in agreement with appropriate guidelines and regulations. The methodology was approved by the institutional ethical committee of Madurai Kamaraj University (Internal research and review board (IRB), ethical clearance (EC), biosafety, and animal welfare committee.

### Isolation and screening of biosurfactant producers from human skin

Skin swabs were collected from healthy individuals with no observable symptomatic skin diseases. The individuals were provided with a sterile swab moistened with Tris–EDTA and 0.5% Tween 20. Retro auricular creases and the Antecubital fossae were swiped vigorously for 30 s with the swabs and samples were introduced into a tube containing transport^27^. The collected samples were serially diluted in 0.85% saline and plated on de Man Rogosa and Sharpe (MRS) medium. After incubation, the individual isolates were evaluated for its acid tolerance (pH 4.5) nature using MRS broth. Human cathelicidin LL-37, a multifunctional host defense peptide was used to screen the true skin bacteria. Simulated skin defense environment was prepared by dissolving LL-37 (500 μg/ml, 111 μM) in PBS^28^. The tolerance towards LL-37 was evaluated from that described by Lebeer et al.^29^. At the end of the incubation of isolates with serial dilutions of each reaction mixture were used to inoculate MRS agar plates. The number of colonies was counted for acid and defense tolerant strains were screened for biosurfactant production. Extracellular anionic biosurfactant production was screened by a drop collapse test, CTAB method, and lipase production. The surfactant activity was quantitatively evaluated by the emulsification index (EI_24_) and surface tension determination. For the molecular identification of the best biosurfactant producer strain, the 16S rRNA region was amplified using the universal primers 27F/1492R^30^. PCR products were purified and sequenced. Phylogenetically linked bacterial rDNA sequences were retrieved from the NCBI GenBank database and the neighbor-joining method was adapted to construct the phylogenetic tree with MEGA 5.0.

### Production, purification, and characterisation of biosurfactant

Culture conditions and biosurfactant preparation were carried out as described by Kannan et al*.*^30^. Acid precipitated biosurfactant was subjected to repeated extractions with an ethyl acetate–methanol mixture (2:1, v/v). The organic phases were collected, combined and condensed in a rotary vacuum evaporator, and weighed. The biosurfactant was subjected to fast protein liquid chromatography (FPLC) on DEAE cellulose column (Bio-Rad) equilibrated with 50 mM Tris (pH 8.2) and 0.1 M NaCl. The surfactant was eluted with the same buffer at a flow rate of 1 ml/min for 60 min and the absorbance was recorded at 280 nm. The fractions exhibiting maximum surfactant activity were pooled, concentrated by lyophilization. The infrared spectrum of the lyophilized biosurfactant was obtained in the absorbance mode at a resolution of 4 cm^−1^ using an FT-IR spectrophotometer (Shimadzu, Columbia, USA). One-dimensional ^1^H nuclear magnetic resonance (NMR) spectra were recorded on a 500 MHz NMR spectrometer (Bruker, Rheinstetten, Germany) with 100% CDCl_3_ (Sigma-Aldrich, India), using 5 mg of the biosurfactant.

### Synthesis of Cu-NP and its characterization

Particles were synthesized by four-step chemical reductions with copper sulphate II pentahydrate as a precursor^31^. The synthesized particles were examined using UV–vis absorption spectroscopy (Jasco UV–Vis V530) in the wavelength range of 450–800 nm. XRD analysis of CuNPs was performed on a diffractometer operated at 40 kV and 30 mA with Cu Ka radiation (1.54 Å) as a source. The scanning range of 2 h was between 10° to 80°. The elemental composition of the nanoparticle was studied by scanning electron microscopy (SEM, JEOL JSM-6490A) armed with an energy-dispersive X-ray spectrometer (EDX) (6490 LA). The particle size and zeta potential of the suspended particles were acquired by Beckman Coulter particle size analyzer through dynamic light scattering. The analysis was carried out at 25 °C and the polydispersity index (PDI) was sustained at 0.33 to ensure the appropriate distribution. Transmission electron microscopic (TEM) investigations were carried out on a JEM-2010 (JEOL) instrument equipped with a slow-scan CCD camera and at an accelerating voltage of 200 kV.

### Liposome preparation and characterization

For the preparation of liposomal lipopeptide biosurfactant abetted CuNPs phosphatidylcholine and cholesterol (2:1 ratio) were taken into the round bottom flask linked to the rotary evaporator and the temperature was set into 37 °C. The phase of evaporation proceeded till the visualization of the dry film. The remaining solvent was recovered by vacuum evaporation for 1 h, subsequently, biosurfactant and CuNPs were added for the synthesis of EL-LP-CuNPs (Encapsulated liposomal lipopeptide abetted CuNPs). The contents were evaporated in the rotatory flask at 60 rpm for 30 min at room temperature and kept stagnantly for 2 h. The non-entrapped biosurfactant and CuNPs were detached by dialysis overnight by 1% DMSO. The particle size, polydispersity index, and zeta-potential of the liposomal preparation were obtained from DLS measurements by using a Beckman Coulter particle size analyzer through dynamic light scattering. For the visualization of liposomal vesicles, TEM investigations were carried out by negative staining with a 2% aqueous solution of phosphotungstic acid. The liposomal preparations were loaded and dried on a carbon-coated grid for staining prior visualization.

### Minimum inhibitory concentration (MIC) determination

The minimal inhibitory concentration (MIC) of biosurfactant alone, CuNPs alone, and EL-LP-CuNPs were evaluated by the broth micro-dilution method using Mueller Hinton broth (MHB)^30^. Varying concentrations of LP, CuNPs, EL-LP-CuNPs were prepared and incubated with the pathogens under definite conditions to assess the MIC. The overnight grown pathogens were aliquoted (1:10) in MHB and grown until exponential phase. 1 × 10^5^(CFU)/ml cells were prepared as inoculum from exponentially grown cells. Serial twofold dilutions of lipopeptide, CuNPs, and EL-LP-CuNPs from 1 to 600 μg/ml were added to the MHB cell suspensions. The microtiter plates were incubated for 16–24 h at 37 °C and the absorbance values of each well were recorded at 600 nm using a Spectramax M3, USA. MIC values were determined from the uppermost dilution showing a visible growth inhibition of the tested strain.

### Killing kinetic assay

Time-kill curve studies were conducted by the method described by Klepser et al*.*^32^ including those outlined in CLSI document M26-A. *P. aeruginosa* ATCC 25,619 and MRSA were subcultured before the experiments were conducted and grown on MHB at 37 °C for 24 h. The inoculum density was adjusted to 0.5 McFarland turbidity standards by spectrophotometrically with OD_530_ nm. LP, CuNPs, and EL-LP-CuNPs were individually added to the prepared cell suspensions at the final drug concentration of MIC and 2 × MIC. A growth control with no drug was also included. The preceding inoculum was assessed immediately after dilution from the growth control tube and measured at zero time as the count. After adding drug combinations, the tubes were incubated with shaking (180 rpm at 37 °C. The viable cell counts were done at 2 h interval up to 24 h by collecting 0.1 ml of the cells, serially diluting and plating in MHA plates. Further, the MHA plates were incubated at 37 °C for 12–18 h. After incubation, the CFU/ml was recorded by counting the number of colonies.

### Inhibition of carotenoid synthesis from P. aeruginosa and MRSA

*P. aeruginosa* cultures were grown overnight and subculture into 1:1,000 dilution in fresh KB medium with the presence of sub MIC range of four dilutions of treatment combinations (LP alone, CuNPs alone, EL-LP-CuNPs) and incubated for 16–18 h. the untreated broth was kept as control. After incubation, the culture was centrifuged and the supernatant was extracted with chloroform and 0.2 M HCL. After extraction, the OD was recorded at 520 nm^33^.

For the quantification of staphyloxanthin, MRSA was inoculated in 5 ml of TSBG supplemented with the sub MIC range of four dilutions of treatment combinations and grown at 37 °C for 24 h at 160 rpm. After incubation, cells were harvested by centrifugation at 10, 000 rpm for 5 min, and the cell pellets were washed twice with PBS. Tubes containing MRSA control and treated cell pellets were visually observed for staphyloxanthin inhibition and photographed. For qualitative analysis of staphyloxanthin inhibition, cell pellets were resuspended in ethanol and incubated at 40 °C for 20 min and centrifuged at 10,000 rpm for 5 min. Then, the collected ethanolic extract was dried under vacuum and the crude staphyloxanthin concentrate was mixed with ethyl acetate/1.7 M aqueous sodium chloride. The organic layer was saved and the ethyl acetate extraction was repeated until the aqueous layer becomes colorless. The presence of carotenoids was measured at 286, 435, 455, and 462 nm, (Spectramax M3, USA)^34^.

### Inhibition of secreted proteases and extracellular polymeric substances (EPS)

For the EPS quantification, ice-cold sulfuric acid (0.2 M, pH 1.1) was added with the biofilm pellet and homogenized with the glass beads to breakdown the film matrix^35^. Further, the entire suspension was stirred for 3 h at 4 °C and centrifuged at 15,000 rpm for 20 min. The supernatant comprises the total EPS of both capsular and colloidal fractions was denoted as the EPS solution. The dry weight of the cell pellet was subtracted from the biofilm dry weight and determined as the EPS dry weight.

## Biofilm metabolic activity—XTT reduction assay

XTT sodium salt and menadione was prepared freshly at the ratio of 12.5:1 in sterile PBS (1 mg ml^−1^) and acetone (1 mM) respectively. After the development of biofilm with and without the treatment combinations, planktonic components of the bacterial growth were detached and eroded twice with sterile PBS. 25 μl of XTT-menadione mixture was added with 200 μl of collected cell suspension and incubated at 30 °C for 1 h. After incubation, the entire reaction mixture was centrifuged at 5000 rpm for 10 min and the OD of the supernatant and their respective blanks were measured at 490 nm.

### Quantification of intracellular ROS (reactive oxygen species) production

Intracellular ROS with the presence of treatment conditions was quantified using a cell-permeable fluorescent probe, 2–7-dichloro dihydrofluorescein diacetate (DCF-DA). The pathogenic bacterial suspensions were adjusted to the OD of 0.5 in PBS and treated with the MIC of LP, CuNPs, and EL-LP-CuNPs for 1 h. The cells were washed with PBS then 5 µg ml^−1^ of DCFDA was added and incubated for 1 h at 37 °C. Subsequently, the cells were washed thrice with PBS and the fluorescence intensity was analyzed by flow cytometer (BD FACS Aria III, BD Biosciences, San Jose).

### Investigation of viability and membrane integrity

The impact of LP, CuNPs and EL-LP-CuNPs on the viability and membrane integrity of *P. aeruginosa* and MRSA was evaluated following the manufacturer's guidelines using the LIVE / DEAD BacLight kit (Invitrogen, USA). The bacterial suspensions were incubated for 4 h with LP, CuNPs and EL-LP-CuNPs at MIC at 37 °C. Then the suspension was stained with SYTO9 vs. propidium iodide (PI) mixes in the ratio of 1:1 and incubated in the dark for 15 min. Then, the fluorescence intensity of live populations (SYTO9) and membrane damaged dead (PI) populations in each drug treatment group were recorded using a flow cytometer. A total of 50,000 events were recorded. The background noise and debris were eliminated by fixing the cell population acquisition gate based on forward scatter (FSC) and side scatter (SSC) channels.

## Biofilm inhibition assays

### Assessment of biofilm biomass

The biofilm inhibition percentage was measured using a quantitative microtiter plate assay with 18 h biofilm as per previous reports with or without treatment combinations^30^. Briefly, the biofilms were grown to stationary phase in 96-well polystyrene flat-bottom microtiter plates (BD Falcon, Sparks, MD, United States) for 18 h at 37 °C. 1/2 MIC of EL-LP-CuNPs, LP alone, CuNPs alone was added at the time of inoculation. Subsequently, the biofilm wells were washed with distilled water to eliminate the planktonic cells. Then, the adhered cells were stained with 0.5 ml of 0.2% crystal violet (CV) solution (HiMedia, India) for 2 min. The excess stain was detached by washing with distilled water and the CV bound cells biofilm were solubilized with the addition of 0.5 ml of 20% glacial acetic acid. The staining was visually assessed and scanned at 570 nm using a 96- well plate spectrophotometer (Spectra Max M3, USA).

### High content screening (HCS) imaging of biofilm

To analyze the effect of EL-LP-CuNPs, LP alone, CuNPs on biofilm formation with the help of high content imaging system ((HCS) Operetta, Perkin Elmer, USA) the biofilms were allowed to form in high content screening plates (Vision Plate 384 wells black sterile, Perkin Elmer, USA) with 200 μl of sterile BHI for 18 h at 37 °C. 1/2 MIC of all the three different treatment combinations were added in each well at the time of inoculation. After the incubation period, the excess medium was removed, washed twice with sterile phosphate buffer (50 mM, pH 7.0) to remove the planktonic cells, and the biofilms were stained with 15 μl acridine orange (5 μg/ml) (Sigma, USA). To determine viable biofilm matrix after exposure to treatment combinations by HCS, the LIVE/ DEAD BacLight Bacterial viability assay (Thermo Fisher, UK) was used. SYTO9 and propidium iodide stock solutions were prepared according to the manufacturer’s instructions. The biofilms were grown in HCS plates for 18 h of incubation and treated with EL-LP-CuNPs, LP alone, CuNPs for 30 min. 15 μl of dye combinations were added in both control and treated wells and incubated in dark for 10 min. The images were taken and Z-stack analysis was done with the Harmony software (Perkin Elmer–USA)^30^.

### Scanning electron microscopic analysis of biofilm

A scanning electron microscope was used to evaluate the consequence of treatment combinations on in vitro biofilm. *P. aeruginosa* and MRSA were statically accustomed in 1 × 1 cm glass slides with 1/2 MIC of treatment combinations for 18 h. The entire setup was incubated in 4 well cell culture plates at 37 °C and the slides were mildly washed, placed in 3% glutaraldehyde fixative (in PBS). The samples were dried in a freeze dryer and coated with gold using an ion coater (IB-5, Eiko, Kanagawa, Japan). Biofilms were visualized by using a Vega 3—Tescan scanning electron microscope and analyzed using Vega 3—Tescan Essence software^30^.

### Bacterial ultra-structural analysis by TEM

*P. aeruginosa* and MRSA cells were exposed to treatment combinations at 1/2 MIC levels for 18 h, fixed in 2.5% glutaraldehyde, 4% paraformaldehyde in 0.1 M cacodylate buffer (pH 7.3) for 30 min. The copper grids were removed and visualized under TEM^30^. The images were analyzed using Tecnai G2 20—Xplore3D software.

### Coating of urinary catheters with EL-LP-CuNPs for the In vitro antibiofilm activity

Medical-grade polyurethane catheters, Rusch Gold Two-Way Silicone Coated Latex Foley Catheter, and Romsons Suction Catheter with Connector were coated with EL-LP-CuNPs (Yao et al., 2008). The coating resulted in a thin layer of EL-LP-CuNPs onto the surface of the catheters. For the in vitro assay, catheters were cut into 1-cm-length portions and immersed in 2 ml of PBS. The seeding of pathogens was done by immersing the catheters containing *P. aeruginosa* and MRSA ATCC 43,300 (10^6^ and 10 ^8^ CFU/ml) for 2 h at 37 °C, with shaking at 100 rpm. The catheters were gently recovered after incubation and one set were placed in 3% glutaraldehyde fixative (in PBS) for the SEM analysis. The remaining sets were washed with PBS to remove the planktonic cells and incubated in 1 ml of trypsin–EDTA for 1 h at 37 °C with gentle shaking (100 rpm). Later the tubes were sonicated at 100 Hz for 5 min and vortexed for 30 s. The entire dislodged suspension was serially diluted and plated on KB medium and Hichrome Aureus agar (Himedia, India).

## Evaluation of in vivo therapeutic potential on C. elegans

### C. elegans strain and culture

Wild-type N2, Bristol strains of *C. elegans* were (obtained from *C. elegans* Genetics Center CGC, University of Minnesota, USA) grown on nematode growth medium (NGM) with bacterial culture *E. coli* OP50 as a feed, at 20 °C for 2 days to obtain gravid adults.

### In-vivo adherence using C. elegans

To examine the in vivo internal colonization microscopically in *C. elegans*, the washed worms were stained with 0.1% Acridine orange for 3 min. the excess acridine orange was removed by extensive washing with M9 buffer and the colonization of *P.aeruginosa* and MRSA in *C. elegans* intestine was observed under HCS. The fluorescent strength of acridine orange in the intestine and surface of *C. elegans* is directly proportionate to the adherence by *P.aeruginosa* and MRSA^36^.

### Survival and toxicity assessment on C.elegans

To investigate the in vivo toxicity and impression of treatment combinations on the virulence of both *P. aeruginosa* and MRSA, the nematode *C. elegans* was infected with the pathogens. LPs alone, CuNPs alone, and EL-LP-CuNPs challenging groups were also included to evaluate the intrinsic toxicity of drug combinations towards *C.elegans*. Synchronized adult *C. elegans* nematodes were then allowed to feed on the *P.aeruginosa* and MRSA lawn with and without LPs, CuNPs, and EL-LP-CuNPs for 4 h at 25 °C. For the killing assay, around 10 worms were transferred to the medium containing biosurfactant and CuNPs at its sub-MICs along with an inoculum of cells of *P.aeruginosa* and MRSA. Finally, the survival of the worms was recorded every 2 h, and worms that did not respond to a touch stimulus by a platinum loop were scored as dead^37^.

### Evaluation of colonization by CFU assay

To determine the colonization by *P. aeruginosa* and MRSA, inside the intestine of *C. elegans*, a pathogen accretion assay was done. Briefly, a lot of 10 nematodes were infected with *P. aeruginosa* and MRSA in the presence and absence of biosurfactant CuNPs and were washed twice with M9 buffer. A countable number (~ 10) of worms were taken in a microcentrifuge tube containing M9 buffer with 1% Triton X-100. The entire mixture was ground mechanically with the sterile micro pestle. Finally, the resulting suspension from *P. aeruginosa* infected worms was serially diluted and plated on KB medium and MRSA infected worms were plated on Hichrome Aureus agar (Himedia, India) to evaluate the CFU. All data point signifies the mean CFU from triplicate trials with standard errors.

### Statistics

All experiments were done in triplicate. All data sets were expressed as arithmetic mean ± standard deviation and analyzed with a two-tailed, paired t-test using Graph-pad prism 7.0.
